# Comparison of the Validity and Generalizability of Machine Learning Algorithms for the Prediction of Energy Expenditure: Validation Study

**DOI:** 10.2196/23938

**Published:** 2021-08-04

**Authors:** Ruairi O'Driscoll, Jake Turicchi, Mark Hopkins, Cristiana Duarte, Graham W Horgan, Graham Finlayson, R James Stubbs

**Affiliations:** 1 Appetite Control and Energy Balance Group School of Psychology University of Leeds Leeds United Kingdom; 2 School of Food Science and Nutrition Faculty of Environment University of Leeds Leeds United Kingdom; 3 Biomathematics & Statistics Scotland Aberdeen United Kingdom

**Keywords:** bioenergetics, energy balance, accelerometers, machine learning, validation

## Abstract

**Background:**

Accurate solutions for the estimation of physical activity and energy expenditure at scale are needed for a range of medical and health research fields. Machine learning techniques show promise in research-grade accelerometers, and some evidence indicates that these techniques can be applied to more scalable commercial devices.

**Objective:**

This study aims to test the validity and out-of-sample generalizability of algorithms for the prediction of energy expenditure in several wearables (ie, Fitbit Charge 2, ActiGraph GT3-x, SenseWear Armband Mini, and Polar H7) using two laboratory data sets comprising different activities.

**Methods:**

Two laboratory studies (study 1: n=59, age 44.4 years, weight 75.7 kg; study 2: n=30, age=31.9 years, weight=70.6 kg), in which adult participants performed a sequential lab-based activity protocol consisting of resting, household, ambulatory, and nonambulatory tasks, were combined in this study. In both studies, accelerometer and physiological data were collected from the wearables alongside energy expenditure using indirect calorimetry. Three regression algorithms were used to predict metabolic equivalents (METs; ie, random forest, gradient boosting, and neural networks), and five classification algorithms (ie, k-nearest neighbor, support vector machine, random forest, gradient boosting, and neural networks) were used for physical activity intensity classification as sedentary, light, or moderate to vigorous. Algorithms were evaluated using leave-one-subject-out cross-validations and out-of-sample validations.

**Results:**

The root mean square error (RMSE) was lowest for gradient boosting applied to SenseWear and Polar H7 data (0.91 METs), and in the classification task, gradient boost applied to SenseWear and Polar H7 was the most accurate (85.5%). Fitbit models achieved an RMSE of 1.36 METs and 78.2% accuracy for classification. Errors tended to increase in out-of-sample validations with the SenseWear neural network achieving RMSE values of 1.22 METs in the regression tasks and the SenseWear gradient boost and random forest achieving an accuracy of 80% in classification tasks.

**Conclusions:**

Algorithms trained on combined data sets demonstrated high predictive accuracy, with a tendency for superior performance of random forests and gradient boosting for most but not all wearable devices. Predictions were poorer in the between-study validations, which creates uncertainty regarding the generalizability of the tested algorithms.

## Introduction

### Background

Participation in physical activity results in increased energy expenditure [1] and represents a key modifiable risk factor for cardiovascular disease, obesity, diabetes mellitus, cancer, and mortality [2]. Thus, longitudinal, unobtrusive, and accurate measurement of intraday physical activity energy expenditure would be highly valuable for health research. Activity trackers offer a scalable means for the continuous collection of physical activity data in free-living environments and, by extension, the measurement of energy expenditure. Unfortunately, the accuracy of activity trackers varies greatly between devices and activities [3,4], which limits their use when quantifying energy balance and activity behaviors.

The potential of machine learning techniques to model the complex interactions of accelerometer data, physiological variables, and the rate of energy expenditure has been recognized for some time. Rothney et al [5] trained an artificial neural network using raw accelerometer data as input to predict the energy expenditure in a whole-body calorimetry chamber. Pober et al [6] used quadratic discriminant analysis and a hidden Markov model to classify activity and subsequently estimated the proportion of time performing different activities. Research groups have built on these early findings and have reported highly accurate algorithms for a variety of activities [7-11]. Researchers often take two broad approaches when modeling physical activities: first, attempting to predict the rate of energy expenditure, and second, classifying a minute as sedentary activity, light physical activity, or moderate-to-vigorous physical activity (MVPA), both of which are important for health research. Regression approaches can be used to derive the total energy expenditure for a subject and this can subsequently be incorporated into energy balance models to calculate energy intake [12]. Alternatively, accurately determining the time an individual spends in broader categories of activity or the intensity of that activity can be important for public health guidance. For example, successful weight maintenance in the National Weight Control Registry and weight management recommendations are often defined based on the time an individual spends in MVPA [13]. Machine learning algorithms have the potential to enhance physical activity assessment beyond that of traditional count-based methods, which despite being more accessible, may not be sufficiently accurate for the assessment of energy expenditure and intensity classifications [14].

Recently, we demonstrated in a laboratory validation study that accelerometer and physiological sensor outputs can be modeled using random forests to predict the rate of energy expenditure (as a multiple of resting energy expenditure) in commercial and research-grade activity monitors. We demonstrated a low error in the prediction of energy expenditure [15]. The number of activities in which energy expenditure was measured in this study was limited, and the generalizability of these algorithms remains uncertain. A method for continued refinement of predictive algorithms is to obtain more than one data set [16] to provide larger, more diverse training data with more activities. More data present a new optimization problem, which (because of different assumptions made by different algorithms) means that there is no guarantee that any algorithm will minimize error on all problems [17]. For machine learning models to be used in general health research settings, it is critical to evaluate the generalizability of prediction algorithms. The extent to which an algorithm will generalize is influenced by the characteristics of the sample, activity types, size, and quality of the training data. One approach that addresses each of these limitations is to evaluate prediction algorithms on different samples using data collected under different conditions. In addition to generalizability, a combination of heterogeneous data sets collected under different experimental conditions may help to increase the accuracy of predictions [18].

### Objectives

In this study, two distinct data sets of concurrent inputs from multiple wearable devices (ie, Fitbit Charge 2, ActiGraph GT3-x, SenseWear Armband Mini, and a polar chest strap) and measured energy expenditure (indirect calorimetry) are combined to develop predictive models of minute-level energy expenditure and physical activity. We aim to evaluate classification and regression algorithms to (1) predict the rate of energy expenditure and (2) classify a single minute as sedentary activity, light physical activity, or MVPA. Algorithms were validated using leave-one-subject-out cross-validation (LOSO) and out-of-sample validation. Concurrently, we evaluated the SenseWear armband, a device that has been shown to outperform accelerometer-based monitors when classifying activity minutes [19] and is one of the most accurate wrist or arm-based monitors for estimating energy expenditure [3].

## Methods

### Studies

This study aggregated the data collected as part of two separate studies at the Human Appetite Research Unit, University of Leeds. Participants were recruited from the local area using word-of-mouth and recruitment emails. Participants must have been at least 18 years of age, have been able to attend the research laboratory at the required intervals, be able to ambulate without assistance, they must not have been taking medications known to alter metabolic rate, and participants must not have had any cardiovascular, metabolic, renal disorders, illness, or injury that would increase the risk of medical events during physical activity. Both studies were approved by the University of Leeds, School of Psychology Ethics Committee (PSC-407 and PSC-744 for study 1 and 2, respectively), and all participants provided informed consent before participation in the study. The participant information for the samples is shown in Table 1. Study 2 had proportionately more males, lower age, lower average percentage of fat mass (FM), and a higher resting metabolic rate (RMR) on average.

**Table 1:** Characteristics of the included sample.

Study	Participants			Age (years), mean (SD)		Height (cm), mean (SD)		Weight^a^ (kg), mean (SD)		FFM^b^ (kg), mean (SD)		FM^c^ (kg), mean (SD)		FM (%), mean (SD)		RMR^d^ (kcal/d), mean (SD)
	Total	Female, n (%)														
1	59	41 (69)	44.4 (14.1)		167.5 (8.9)		75.7 (13.6)		49.8 (8.9)		24.8 (10.7)		32.5 (10.3)		1581.8 (280.4)	
2^a^	30	13 (43)	31.9 (10.2)		171.9 (9.2)		70.6 (12.9)		55 (12.6)		15.1 (7.1)		21.7 (8.7)		1769.3 (435.8)	

^a^In study 2, resting metabolic rate and body composition were estimated at a subsequent visit to the laboratory and therefore weight is not the sum of fat mass and fat-free mass; in study 1, body composition was not available for all subjects and therefore weight is not the sum of fat mass and fat-free mass.

^b^FFM: fat-free mass.

^c^FM: fat mass.

^d^RMR: resting metabolic rate.

### Protocols

#### Study 1

The details of study 1 have been published previously [15]. The protocol of study 1 consisted of 10 activities, each performed for 5 minutes in the following order: sitting, standing, treadmill walking and incline walking (4 km/h), jogging, and incline jogging (6-8 km/h). Participants then rested for 3 minutes and transitioned to a cycle ergometer for low- and moderate-intensity cycling. After another period of recovery, participants performed a folding and sweeping task. Owing to a variation in physical fitness, the jogging task (n=49), incline jogging (n=30), and moderate cycling tasks (n=58) were not performed by all participants.

#### Study 2

In study 2 (total energy expenditure from wearable devices study), participants visited the lab and refrained from eating or consuming caffeine for at least 4 hours. This exercise visit is the first of three visits to the laboratory conducted as part of a wider project. Weight and height were obtained from a SECA 704s stadiometer and electronic scale (SECA, Germany), and subsequently, an activity protocol was performed. All activities were performed in 5-minute increments, and the order was identical for all participants. First, resting tasks were performed where participants lay supine, sat in a backed chair, and then stood. Next, after a 2-minute unstructured transitional period, participants performed seated typing, standing ironing, and wiping surfaces while standing. After another 2-minute transition, participants walked on a treadmill at 4 km/h, walked at an incline of 5% at 4 km/h, and subsequently jogged at 7 km/h. The participants then rested for 10 minutes. After the unstructured resting period, participants performed low-intensity and moderate-intensity cycling, low-intensity and moderate-intensity rowing, and low-intensity and moderate-intensity cross-training (elliptical), with 1-minute transitions between each, and the intensity of the tasks was determined by a self-selected perceived exertion. In study 2, one participant did not perform rowing or elliptical tasks.

### Body Composition Assessment

In both studies, body composition was estimated using air displacement plethysmography (BodPod, Life Measurement, Inc), n=57 in study 1 and n=30 in study 2. Study 2 is part of a wider study in which participants visited the laboratory three times, the first of which was the laboratory validation reported here. Body composition was measured at a subsequent visit to the laboratory in a fasting state.

### Energy Expenditure

This study used metabolic equivalents (METs) as the outcome variable, which served to eliminate the proportion of energy expenditure attributable to RMR. We first established the RMR of each participant, which was measured in the fasting state, before any exercise. In both studies, RMR was determined from VO_2_ and VCO_2_ data collected through a ventilated hood indirect calorimeter system (gas exchange measurement; Nutren Technology Ltd). In study 1, RMR was measured before exercise testing, and in study 2, which occurred on a subsequent visit to the laboratory. After researchers explained the procedures to the participants and an initial calibration process (approximately 10 minutes), VO_2_ and VCO_2_ were measured for 30 minutes in the supine position. The RMR was established from the VO_2_ and VCO_2_ of the 5-minute block with the lowest coefficient of variation [20]. If RMR data were unavailable (n=3 across both studies), we approximated the RMR with BMI-specific equations [21]. During the activity sessions, energy expenditure was obtained from a stationary metabolic cart (Vyntus CPX, Jaeger-CareFusion), and these data were expressed relative to the measured RMR of each subject to derive METs. Definitions of METs are inconsistent [22] and we took an individualized approach to METs calculations because the *standard* definition of METs may have limited applicability in some subjects [23].

### Devices

Accelerometer and physiological data were collected using various sensors in both protocols. The Polar H7 chest strap (Polar Electro) was used to measure the heart rate. An ActiGraph GT3-X accelerometer (ActiGraph) and a Fitbit Charge 2 (Fitbit Inc) were attached securely to the nondominant wrist. Participants also wore the SenseWear Armband Mini (BodyMedia Inc) on the upper arm.

### Data Aggregation

The sensor outputs were obtained from the device-specific software and aggregated to the minute level and time matched to the criterion energy expenditure data. Data loss attributable to device malfunction was as follows: in study 1, Fitbit data of 2 participants, ActiGraph data of 1 participant, and polar heart rate data of 1 participant were lost. In study 2, 1 SenseWear and 1 Fitbit data set were lost because of device failure. Given the slightly different data availability in each model, our results report the number of minutes used and the number of participants. All minutes in which energy expenditure data were available (ie, face mask was not removed) were included in this analysis, and the aggregation of the data sets by time was conducted in Python 3.7.6 and R version 3.6.3 (R Core Team).

For activity-specific analyses, we grouped activities into broader categories. *Activities of daily living,* which involved folding, sweeping, typing, ironing, and wiping surfaces. Distinct categories were assigned for *cycling*, *elliptical*, *rowing*, *running*, and *walking*. The sedentary activities involved all sitting, standing, and supine tasks. The transitional category refers to unstructured resting or transitional minutes.

### Features

Predictive models were built for Fitbit, ActiGraph, and SenseWear, and the features used in each model are listed in Table 2. Each device used a combination of subject-level features, accelerometer features, and physiological features, which have been related to the rate of energy expenditure in previous studies [3,5,24-26]. The features varied depending on the feature availability of each device. Where small (limit of 5 minutes) heart rate gaps existed (eg, loss of signal between the respective heart rate sensor and the skin), we used linear interpolation to fill gaps. As activity in the preceding minutes influences the rate of energy expenditure at the measurement point [27], some time-lagged features were computed: for steps (Fitbit and SenseWear), vector magnitude (ActiGraph), Fitbit heart rate (Fitbit), and polar heart rate (SenseWear and ActiGraph), the change from t-1 minutes for each minute up to t-5 minutes were included as predictive features. In addition, the mean and SD of the current and last 5 minutes were used as predictive features. If time-lagged variables could not be computed due to missing data (ie, for the first minutes for each subject), we imputed backward using the next available observation.

As a constant variance is important for some of the algorithms tested in this study, all numeric features were standardized before training using the following formula:

z = (x – μ) / sd

**(1)**

where *μ* and *sd* refer to the variable mean and SD, respectively.

**Table 2:** Predictive features used in each of the models.

Device^a^ and category		Features
**Fitbit**		
	Subject features	Gender, age, height, weight, and sitting heart rate
	Acceleration features	Steps features:
		steps mean, steps difference (t-1, t-2, t-3, t-4, and t-5 minutes); steps mean and SD of last 5 minutes
	Physiological features	Fitbit heart rate features:
		Fitbit heart rate above sitting heart rate, Fitbit heart rate percentage of maximum heart rate, Fitbit heart rate mean, Fitbit heart rate difference (t-1, t-2, t-3, t-4, and t-5 minutes), and Fitbit heart rate mean and SD of last 5 minutes
**ActiGraph**		
	Subject features	Gender, age, height, and weight
	Acceleration features	X, Y, Z features: minimum, maximum, mean, SD; median crossings; 10th, 25th, 50th, 75th, 90th percentiles; correlations (XY, XZ, YZ); dominant frequency; dominant frequency magnitude First order differential of X, Y, Z features: minimum, maximum, mean, SD; median crossings; 10th, 25th, 50th, 75th, and 90th percentiles; correlations (XY, XZ, YZ); dominant frequency; dominant frequency magnitude Vector magnitude features: vector magnitude mean; vector magnitude difference (t-1, t-2, t-3, t-4, and t-5 minutes); vector magnitude mean and SD of last 5 minutes
	Physiological features	Polar heart rate features: polar heart rate above sitting heart rate; polar heart rate percentage of maximum heart rate; polar heart rate mean; polar heart rate difference (t-1, t-2, t-3, t-4, and t-5 minutes); polar heart rate mean and SD of last 5 minutes
**SenseWear**		
	Subject features	Gender, age, height, and weight
	Acceleration features	X, Y, Z features: peaks, mean of absolute differences, average; Steps features: steps mean; steps difference (t-1, t-2, t-3, t-4, and t-5 minutes); steps mean and SD of last 5 minutes
	Physiological features	Polar heart rate features: polar heart rate above sitting heart rate; polar heart rate percentage of maximum heart rate; polar heart rate mean; polar heart rate difference (t-1, t-2, t-3, t-4, and t-5 minutes); polar heart rate mean and SD of last 5 minutes; and SenseWear sensors: near body temperature average, Galvanic skin response average, skin temperature average

^a^For each device, the subject characteristics, acceleration features, and physiological features are listed.

### Algorithms

The SenseWear outputs a MET estimate that we evaluated in this study (SenseWear manufacturer). We also tested several machine learning algorithms for regression and classification tasks, which are described below. In the regression tasks, algorithms predicted a MET value for each minute, and in the classification tasks, algorithms classified activity categories for each minute. The activity classifications were as follows: sedentary activity (≤1.5 METs), light physical activity (>1.5 and <3 METs), and MVPA (≥3.0 METs) [18,28,29]. For each algorithm, the hyperparameters were informed by a random search through a range of potential hyperparameters in the preliminary tuning experiments. Random search iterates over a grid of randomly selected combinations of hyperparameters, rather than exploring every possible combination of features, and therefore offers a significant computational advantage over a grid-search approach [30]. Each random search was conducted with the RandomizedSearchCV class in Scikit Learn [31], using three-fold cross-validation. The specific parameters for each algorithm are detailed in Multimedia Appendix 1, and except for the neural network models (explained in the following section), the scoring or loss criterion was the default loss or scoring metrics within Scikit Learn. All algorithms were trained using Keras-GPU [32] or Scikit Learn [31].

### Random Forest

The random forest algorithm was used for regression and classification tasks [33]. Random forests involve training of multiple decision trees on data subsamples. Importantly, when splitting these decision trees, only a subsample of the potential predictors is used, which serves to *decorrelate* the trees. The predictions of each tree can then be combined to produce a majority vote (classification) or continuous prediction (regression). The optimal hyperparameters of the algorithm were estimated in the tuning experiments and included the number of trees, number of samples required to split a tree, number of samples per leaf, total predictors, and the depth of trees. In regression, the quality of a split was assessed with mean square error, and in classification, Gini impurity was used. Algorithms were implemented using the *RandomForestClassifier* and *RandomForestRegressor* classes in Scikit Learn [31].

### Gradient Boosting

For the regression and classification tasks, we used the gradient boosting algorithm. Similar to random forests, this algorithm is a tree-based ensemble method. However, where random forests may be considered to use a *bagging* approach, gradient boosting uses *boosting* to learn. Boosting involves the sequential growth of small (weak) decision trees. Each tree is trained using the residuals of the previous estimator and subsequently added to the fitted function to update the residuals. In the boosting phase, a learning rate parameter penalizes the contribution of each tree to the overall model, thereby slowing the learning [34]. The gradient boosting hyperparameters were tuned in the random search experiments and included the number of boosting stages, the maximum depth of the estimators, learning rate, number of samples required to split a node, the number of samples per leaf, and the maximum number of predictors. In the regression, the loss function was least squares, and in classification, deviance was used. Algorithms were implemented using the *GradientBoostingClassifier* and *GradientBoostingRegressor* classes in Scikit Learn [31].

### Neural Networks

The third algorithm, used in both regression and classification tasks, was artificial neural networks. Neural networks allow complex, nonlinear functions to be modeled and comprise layers of interconnected *neurons*. At each neuron, inputs are subjected to a numerical activation function, and then passed through subsequent hidden layers of neurons to an output layer [34,35]. In the training process, the interneuronal weights of the network are refined relative to a loss function (ie, mean square error or cross-entropy). Neural networks in the classification studies used the sparse categorical cross-entropy loss function, and in the regression setting, the loss was the mean square error. We tuned the learning rate of each network, the number of layers, and the number of neurons. Neural networks hidden layers used the *relu* activation function, and classification models used a *softmax* activation in the output layer, both classification and regression networks used the Adam optimizer.

### K-Nearest Neighbors

For classification tasks, we tested the k-nearest neighbor (KNN) algorithm. This algorithm assigns a given point to a particular class based on the majority class of the k nearest neighbors, where the neighbors of a given point are defined by a distance metric (ie, Euclidian, Minkowski, or Manhattan) [34]. Hyperparameters adjusted in the training process included the number of neighbors in each neighborhood (k), distance metrics, and the weight applied to each of the observations in a neighborhood. KNN was implemented with Scikit Learn [31], using the *KNeighborsClassifier* class.

### Support Vector Machine

The final classification model tested was a support vector machine classifier with a radial basis function [35]. A support vector machine aims to find a separating hyperplane between classes by maximizing the distance between the points and the hyperplane. In this study, we tuned the regularization parameter (C) and gamma, which defines the magnitude of the effect of specific training examples. The support vector machine classifier was implemented with the *SVC* class in Scikit Learn [31].

### Statistical Analyses

We conducted two validation approaches for all the analyses and algorithms. First, LOSO validations, where algorithms are trained on all but the data of 1 participant, and the participant is held back for validation. This process was repeated until all participants had served as the validation participant once. Second, we used an out-of-sample validation in which the entire data set from one study was used as training data, and the second study was used as an out-of-sample validation. Regression algorithms were evaluated by root mean square error (RMSE), mean absolute percentage error (MAPE) with the *Metrics* package in R and concordance correlation coefficient (CCC) with *DescTools*. Agreement statistics were calculated at the minute level; however, for visualization purposes, we computed the RMSE at the level of individuals and plotted these values. Equivalence tests were used to determine if the true METs and predicted METs were statistically equivalent; tests used equivalence bounds of 10%, and to be considered equivalent, the 90% CI must fall within the equivalence bounds. Finally, linear mixed models with a random intercept of subject ID were used to investigate differences in RMSE between the models. Comparisons were conducted using the Lme4 [36] package in R, with *P* values adjusted by the Bonferroni method in post hoc comparisons. For classification tasks, we report the *κ* statistic, which compares the accuracy of the predictions to that of a random system. We also report accuracy, where accuracy is the proportion of cases that were classified correctly and the F1 score. All classification statistics were calculated using the Caret [37] package in R. A *P* value of <.05 was used to determine statistical significance, where *P* values were reported.

## Results

### Regression

A total of 89 participant activity sessions were included in this sample, and all models could be evaluated on at least 5448 minutes of data in the LOSO validations.

**Figure 1:**
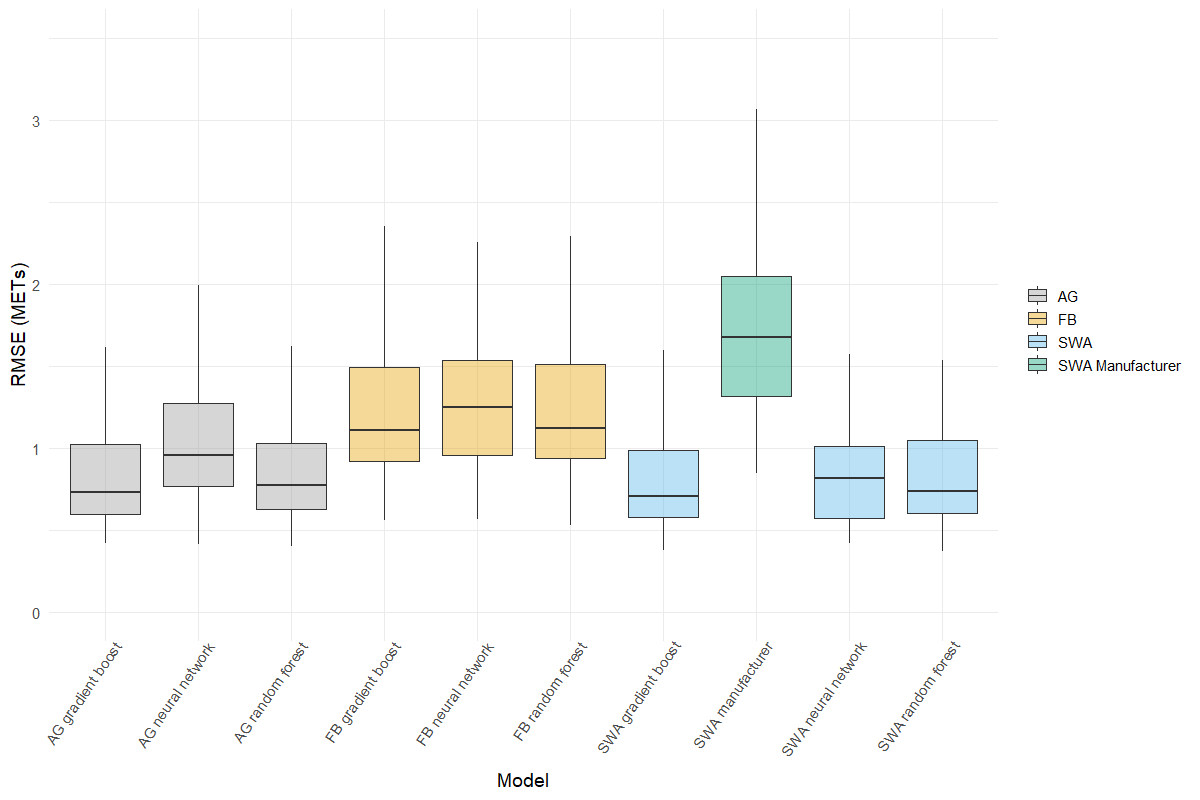
Boxplots demonstrating the root mean square error overall for each of the tested models. AG: ActiGraph; FB: Fitbit; RMSE: root mean square error; SWA: SenseWear.

The regression algorithms predicting energy expenditure are presented for minute-level data in Table 3 and are visually displayed in Figure 1. Our results demonstrate that the greatest error in METs was observed for the manufacturer-provided SenseWear estimates, with MAPE and RMSE values of 34.54 and 1.86, respectively. For ActiGraph, the RMSE was lowest for gradient boosting (0.93 METs), which also achieved the lowest MAPE of any ActiGraph model (17.88%). Of the Fitbit models, the random forest and gradient boosting had equal RMSE (1.36 METs), but a slightly lower MAPE was achieved by the random forest. For the SenseWear, the gradient boost had the lowest RMSE value (0.91 METs), and this was the lowest RMSE of all those tested. The neural network models were associated with a greater overall RMSE for the ActiGraph, Fitbit, and SenseWear models.

**Figure 2:**
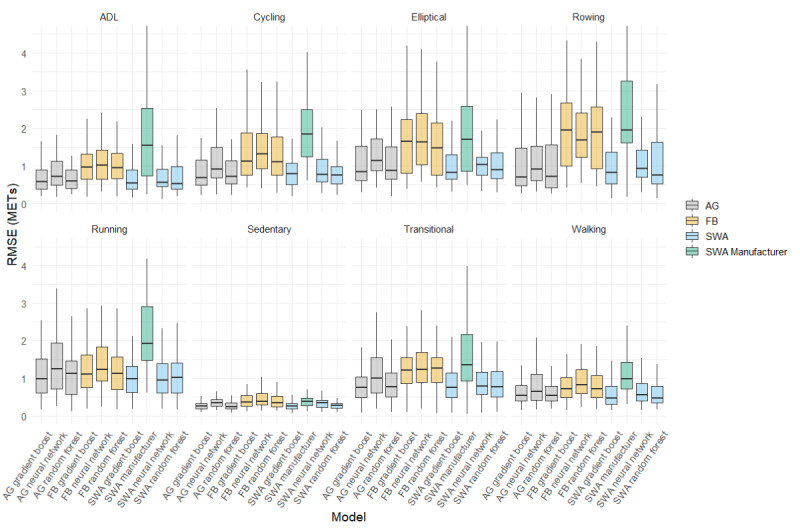
Boxplots demonstrating the root mean square error for each of the tested models in specific activity categories. ADL: activities of daily living; AG: ActiGraph; FB: Fitbit; RMSE: root mean square error; SWA: SenseWear.

**Figure 3:**
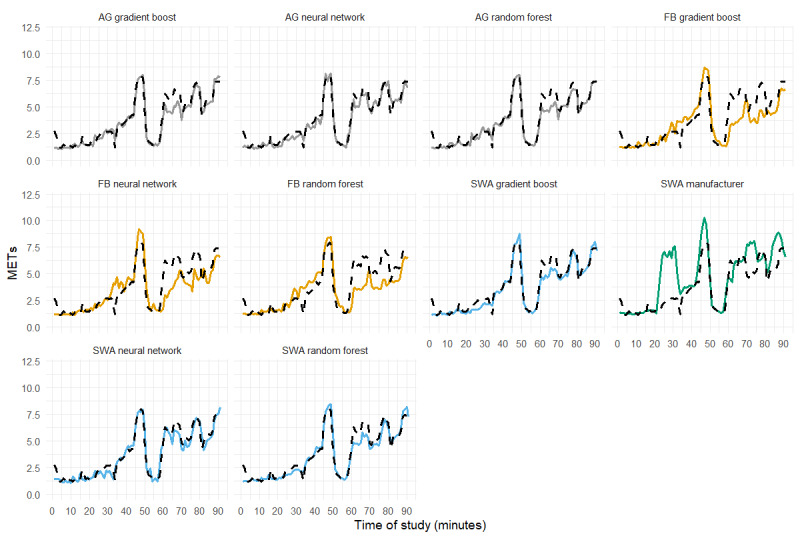
A time series plot showing metabolic equivalents predicted by the models tested in this study (colored solid line) and indirect calorimeter (black dashed line), for a single subject in study 2. The x-axis represents the measurement time. Minutes 1-15=sedentary; minutes 16-17=transitional/unstructured; minutes 18-32=activities of daily living (typing, wiping surfaces, and ironing); minutes 33-34=transitional/unstructured; minutes 35-44=walking; minutes 45-49=running; minutes 50-59=transitional/unstructured; minutes 60-69=cycling; minutes 71-80=rowing; and minutes 82-91=elliptical. Participants performed cycling, rowing, and elliptical tasks at self-selected low and moderate intensity for 5 minutes each. AG: ActiGraph; FB: Fitbit; METs: metabolic equivalents; SWA: SenseWear.

Activity-specific MET predictions are presented in Multimedia Appendix 2, and the RMSE is shown in Figure 2. For all activities tested, tree-based models (gradient boost or random forest) applied to ActiGraph or SenseWear data were superior, as measured by RMSE. The manufacturer estimates of SenseWear had the highest RMSE for all activities aside from sedentary activities, in which only the ActiGraph gradient boost and random forest had a lower RMSE. Notably, all Fitbit models overestimated sedentary activities and had the highest RMSE in this category. The pairwise comparisons between models are presented in Multimedia Appendix 3 for each of the comparisons shown in Figure 1 and Figure 2. An example of the model predictions for a single subject is shown in Figure 3.

Table 4 shows the statistics for the between-study predictions. Notably larger errors were observed relative to the LOSO validations, with the Fitbit gradient boost reaching a RMSE of 1.92 METs (neural network) when study 1 was used as the training data. To estimate the relative importance of each of the features used in each model, permutation importance has been reported in Multimedia Appendix 4.

**Table 3:** Leave-one-subject-out cross-validation results for each of the regression models.

Model	Minutes^a^	Participants, n (%)	Predicted (METs^b^), mean (SD)	True (METs), mean (SD)	MAPE^c^	RMSE^d^	CCC^e^ (95% CI)	Equivalence
SWA^f^ manufacturer	5533	88 (99)	3.8 (2.49)	4.04 (2.59)	34.54	1.86	0.73 (0.72-0.74)	—^g^
AG^h^ gradient boost	5517	87 (98)	4.04 (2.35)	4.04 (2.59)	17.88	0.93	0.93 (0.93-0.93)	Equivalent^i^
AG neural network	5517	87 (98)	4.05 (2.55)	4.04 (2.59)	21.65	1.14	0.9 (0.9-0.91)	Equivalent
AG random forest	5517	87 (98)	4.05 (2.32)	4.04 (2.59)	18.36	0.94	0.93 (0.92-0.93)	Equivalent
FB^j^ gradient boost	5448	86 (97)	4.03 (2.19)	4.01 (2.58)	30.22	1.36	0.84 (0.83-0.84)	Equivalent
FB neural network	5448	86 (97)	4.02 (2.28)	4.01 (2.58)	32.27	1.45	0.82 (0.82-0.83)	Equivalent
FB random forest	5448	86 (97)	4.03 (2.14)	4.01 (2.58)	30.10	1.36	0.84 (0.83-0.84)	Equivalent
SWA gradient boost	5492	87 (98)	4.04 (2.39)	4.04 (2.6)	17.83	0.91	0.93 (0.93-0.94)	Equivalent
SWA neural network	5492	87 (98)	4.05 (2.47)	4.04 (2.6)	19.56	0.96	0.93 (0.92-0.93)	Equivalent
SWA random forest	5492	87 (98)	4.05 (2.35)	4.04 (2.6)	18.25	0.92	0.93 (0.93-0.93)	Equivalent

^a^Minutes refers to the number of minutes the algorithms are validated on.

^b^METs: metabolic equivalents.

^c^MAPE: mean absolute percentage error.

^d^RMSE: root mean square error.

^e^CCC: concordance correlation coefficient CCC is presented with 95% CIs.

^f^SWA: SenseWear.

^g^The model is not statistically equivalent to the criterion.

^h^AG: ActiGraph.

^i^Equivalent implies that the model is statistically equivalent to the criterion.

^j^FB: Fitbit.

**Table 4:** Out-of-sample results for each of the regression models.

Model	Training data	Minutes^a^	Predicted (METs^b^), mean (SD)	True (METs), mean (SD)	MAPE^c^	RMSE^d^	CCC^e^ (95% CI)	Equivalence
AG^f^ gradient boost	Study 1	2690	4.03 (1.9)	3.93 (2.66)	36.35	1.37	0.82 (0.81-0.83)	Equivalent^g^
AG neural network	Study 1	2690	4.07 (2.48)	3.93 (2.66)	29.75	1.33	0.87 (0.86-0.88)	Equivalent
AG random forest	Study 1	2690	3.97 (1.79)	3.93 (2.66)	39.50	1.51	0.78 (0.77-0.79)	Equivalent
FB^h^ gradient boost	Study 1	2630	3.76 (1.7)	3.88 (2.65)	47.55	1.89	0.64 (0.62-0.66)	Equivalent
FB neural network	Study 1	2630	3.65 (1.86)	3.88 (2.65)	47.40	1.92	0.65 (0.63-0.67)	—^i^
FB random forest	Study 1	2630	3.76 (1.66)	3.88 (2.65)	47.45	1.87	0.64 (0.63-0.66)	Equivalent
SWA^j^ gradient boost	Study 1	2633	3.92 (2.13)	3.94 (2.68)	27.35	1.23	0.87 (0.86-0.88)	Equivalent
SWA neural network	Study 1	2633	3.88 (2.26)	3.94 (2.68)	27.07	1.22	0.88 (0.87-0.89)	Equivalent
SWA random forest	Study 1	2633	3.91 (2.07)	3.94 (2.68)	29.54	1.28	0.86 (0.85-0.87)	Equivalent
AG gradient boost	Study 2	2827	4.46 (2.14)	4.15 (2.52)	31.49	1.36	0.83 (0.82-0.84)	—
AG neural network	Study 2	2827	4.24 (2.56)	4.15 (2.52)	29.00	1.42	0.84 (0.83-0.85)	Equivalent
AG random forest	Study 2	2827	4.45 (2.1)	4.15 (2.52)	31.47	1.38	0.82 (0.81-0.84)	—
FB gradient boost	Study 2	2818	4.11 (2.06)	4.13 (2.51)	34.38	1.66	0.74 (0.72-0.75)	Equivalent
FB neural network	Study 2	2818	4.01 (2.04)	4.13 (2.51)	33.10	1.56	0.77 (0.75-0.78)	Equivalent
FB random forest	Study 2	2818	4.21 (2.04)	4.13 (2.51)	33.79	1.62	0.75 (0.73-0.77)	Equivalent
SWA gradient boost	Study 2	2859	4.15 (2.13)	4.14 (2.51)	24.90	1.25	0.86 (0.85-0.87)	Equivalent
SWA neural network	Study 2	2859	3.94 (2.36)	4.14 (2.51)	25.65	1.25	0.87 (0.86-0.88)	Equivalent
SWA random forest	Study 2	2859	4.2 (2.13)	4.14 (2.51)	25.72	1.26	0.85 (0.84-0.86)	Equivalent

^a^Minutes refers to the number of minutes the algorithms are validated on.

^b^METs: metabolic equivalents.

^c^MAPE: mean absolute percentage error.

^d^RMSE: root mean square error.

^e^CCC: concordance correlation coefficient CCC is presented with 95% CIs.

^f^AG: ActiGraph.

^g^Equivalent implies that the model is statistically equivalent to the criterion.

^h^FB: Fitbit.

^i^The model is not statistically equivalent to the criterion.

^j^SWA: SenseWear.

### Classification

**Figure 4:**
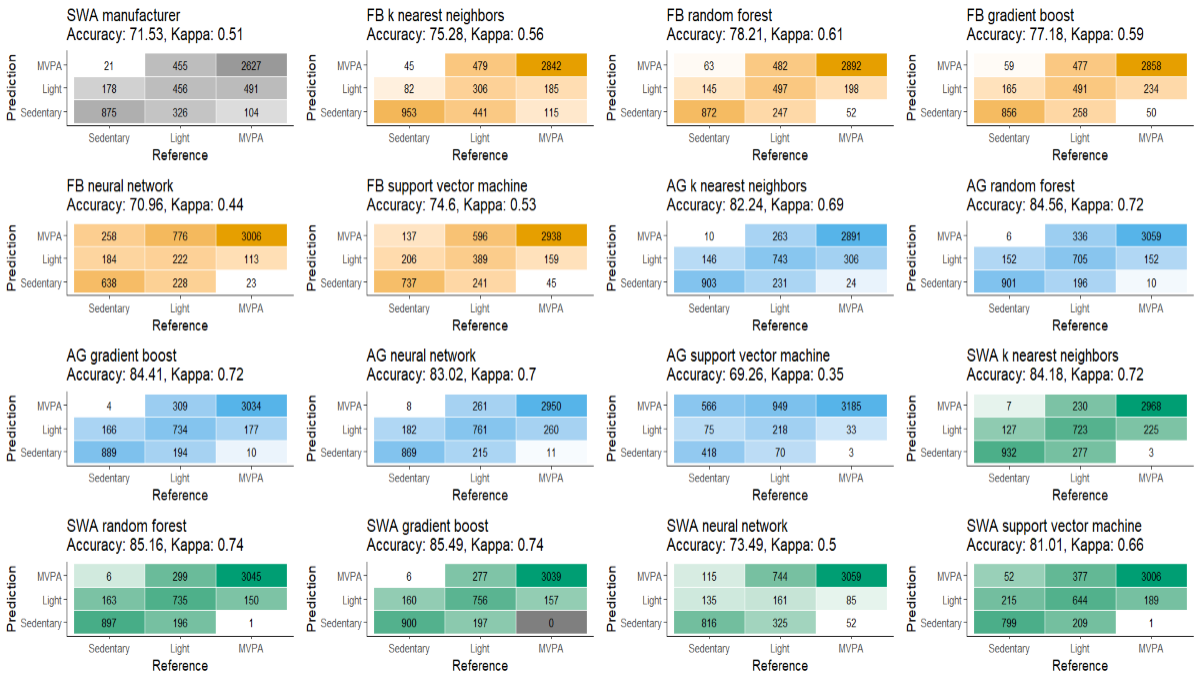
A confusion matrix detailing the classification accuracies for each of the tested models. AG: ActiGraph; FB: Fitbit; SWA: SenseWear.

Figure 4 presents the results of the LOSO classification experiments for all classification algorithms and the SenseWear manufacturer estimates. Classes were slightly imbalanced, approximately 19.4% sedentary activity, 22.4% light physical activity, and 58.2% MVPA with small differences between devices due to data availability. The highest accuracy for Fitbit models was the random forest (78.21%), for the ActiGraph models, the random forest achieved the highest accuracy (84.56%), and for the SenseWear models, the gradient boosting algorithm (85.49%) was the most accurate.

Multimedia Appendix 5 provides class-specific statistics for each model. Models tended to perform worse in light activity with F1 scores ranging from 0.20 (SenseWear neural network) to 0.66 (SenseWear gradient boost). In sedentary activities, the F1 score was improved with a range of 0.54 (Actigraph support vector machine) to 0.83 (four models). For MVPA, the F1 score ranged from 0.80 (Actigraph support vector machine) to 0.93 (three models).

### Between-Study Predictions

The between-study classification accuracies are listed in Table 5. In most cases, when study 1 served as the training data, lower accuracy was observed. When study 1 served as the training data, the accuracy ranged from 0.55 (ActiGraph support vector machine) to 0.80 (two models). When study 2 served as the training data, the accuracy ranged from 0.65 (ActiGraph support vector machine) to 0.79 (three models).

**Table 5:** Between-study classification results for each of the classification models.

Training data and model		Accuracy	*κ*
**Study 1**			
	AG^a^ gradient boost	0.75	0.55
	AG k-nearest neighbors	0.61	0.35
	AG neural network	0.72	0.52
	AG random forest	0.74	0.53
	AG support vector machine	0.55	0.06
	FB^b^ gradient boost	0.67	0.43
	FB k-nearest neighbors	0.68	0.47
	FB neural network	0.67	0.47
	FB random forest	0.67	0.41
	FB support vector machine	0.67	0.45
	SWA^c^ gradient boost	0.80	0.67
	SWA k-nearest neighbors	0.74	0.57
	SWA neural network	0.79	0.66
	SWA random forest	0.80	0.66
	SWA support vector machine	0.68	0.43
**Study 2**			
	AG gradient boost	0.79	0.56
	AG k-nearest neighbors	0.72	0.48
	AG neural network	0.75	0.51
	AG random forest	0.79	0.57
	AG support vector machine	0.65	0.07
	FB gradient boost	0.73	0.48
	FB k-nearest neighbors	0.72	0.47
	FB neural network	0.71	0.44
	FB random forest	0.73	0.48
	FB support vector machine	0.73	0.48
	SWA gradient boost	0.78	0.57
	SWA k-nearest neighbors	0.76	0.55
	SWA neural network	0.76	0.55
	SWA random forest	0.79	0.58
	SWA support vector machine	0.78	0.55

^a^AG: ActiGraph.

^b^FB: Fitbit.

^c^SWA: SenseWear.

## Discussion

### Principal Findings

This study aggregated two laboratory data sets to build on previous work demonstrating the potential for machine learning algorithms to produce accurate estimates of METs and intensity classes in a diverse set of activities and participants. In both regression and classification settings, we observed the smallest errors in energy expenditure predictions when applying tree-based algorithms (ie, random forest and gradient boosting) to SenseWear and ActiGraph outputs with the RMSE and classification errors generally being higher for Fitbit models. In almost all cases, the error was smaller than the SenseWear manufacturer estimates, and in out-of-sample generalizability experiments, we observed greater error and lower accuracy when compared with the LOSO validations. We believe that this is the first study to classify the intensity of activity using machine learning algorithms in Fitbit devices. In Fitbit models, we demonstrated accuracies up to approximately 78% (*κ*=0.6), with superior performance observed for sedentary activity and MVPA classifications, but these were generally less accurate than ActiGraph and SenseWear models, where up to approximately 85% accuracy (*κ*=0.74) was achieved. Taken together, and if these results are verified in free-living, ecologically valid examples, these findings imply that highly accurate estimates of energy expenditure, sedentary activity, and MVPA behaviors can be estimated by the wearables tested here.

### Algorithm Accuracy

We used neural networks, random forests, and gradient boosting in regression tasks. In previous studies, neural networks and random forests have been shown to be effective in modeling energy expenditure [8,9], and our results confirm this to an extent. The RMSE values observed in the trained models ranged from 0.91 METs to 1.45 METs, which improve upon the SenseWear manufacturer value of approximately 1.86 METs. However, when the average METs in this study were considered (approximately 4 METs), it was evident that the energy expenditure prediction could be further improved. It is noteworthy that neural networks resulted in the highest RMSE for all 3 devices and performed particularly poorly for Fitbit models. Similarly, Kate et al [38] showed that neural networks resulted in bias significantly different from 0, compared with bagged decision trees and numerous other algorithms, which were not statistically different. Despite the utility of deep neural networks to model highly nonlinear functions, in some use cases, the *no free lunch* theorems broadly state that there will not be an optimal algorithm for all tasks [17]. Indeed, for our data sets, tree-based ensemble models are generally superior for both learning tasks. It may be that a higher RMSE can be reduced by larger training sets [39].

We generated lagged accelerometer and heart rate variables for each model because the rate of energy expenditure depends on the rate of work in preceding minutes [27], and the relative importance of these metrics is evidenced in the variable importance analyses. Including time-lagged features allows for a clearer distinction between minutes that are relatively similar in their accelerometer pattern but differ in their measured energy expenditure, that is, sitting for a prolonged period versus sitting immediately after running. Transitional minutes were on average approximately 3 METs (largely attributable to the activity in the preceding minutes), compared with sedentary minutes, which averaged approximately 1.3 METs, yet the error statistics were generally comparable with those observed in sedentary minutes, indicating that algorithms could distinguish between those minutes. More advanced neural network architectures (ie, recurrent neural networks) [40] may further the ability of models to capture the temporal dependencies of energy expenditure.

### Generalization

Although many studies have reported low errors when using machine learning approaches in the estimation of energy expenditure or classification of activity, external (out-of-sample) validations are rarer and the opportunity to identify cases of overfitting has been limited. Therefore, we used an out-of-sample validation between the two data sets. In all cases, we observed performance degradation when compared with the LOSO validations. Some of this reduction in accuracy is probably attributable to differences in protocols, activities, and participants, which means that algorithms do not have *similar* minutes on which to train. In addition, it is possible that the algorithms overfit the data. Overfitting occurs when a complex model learns the *noise* in the training data, which does not represent the true underlying function between the inputs and the output [41]. Previous studies have used out-of-sample validation or validation in free-living environments [10,42,43], and when compared with laboratory validations, errors may increase. Concerning the classification of physical activity intensity in multiple samples, a previous study reported reductions in out-of-sample accuracy relative to the within-sample validated models, in some algorithm and data set comparisons [44]. However, the machine learning models still outperformed the Euclidean norm minus one GGIR classification method in out-of-sample testing. In another comprehensive generalizability study, five lab-based heterogeneous data sets were used to predict exercise intensity. This study found that when models were applied to a different data set than those they were generated on, model accuracy decreased from 72-95% to 41-60% [18]. These drops are notably higher than those in this study, and this is probably attributable to the greater differences in the accelerometer models, wear position, and samples across the five data sets. However, caution must be exercised in a comparison between studies, as the balance of classes is likely to differ and therefore influence some evaluation metrics.

### Classification

Our LOSO validations demonstrated a relatively high predictive accuracy (75-85%). However, research-grade device models (ActiGraph and SenseWear) were superior. Fitbit devices provide estimates of time in each category (ie, sedentary, light, and MVPA), but the criteria and algorithms remain proprietary. Feehan et al [45] compared estimates of time in intensities with devices such as ActiGraph and Actical, and concluded that more than 80% of studies reported errors >10% with mean differences ranging between 44% and 632% for estimations of activity above light intensity. Importantly, the devices used for comparison in many studies have varying cut points and are not necessarily gold standards. Our results indicate that the application of machine learning to intensity classification can refine the large errors observed in previous studies. Despite the promising results, we emphasize that laboratory studies have limited ecological validity, and future research should seek to address this. Whole-room indirect calorimetry would likely allow more realistic behaviors to be studied while providing a gold standard comparator.

### Strengths and Limitations

A strength of this study is the aggregation of two data sets to provide a more comprehensive and variable data set on which to train models, although the measures (sensors and indirect calorimetry) were the same between studies. The tested cohorts differed demographically, and the protocols were heterogeneous, which provides a good estimate of the applicability of the tested models. Combining data sets also leads to a larger number of participants (n=89), which is a larger sample size than much of the previous literature [7,9,10,44,46,47]. In general, an increase in training observations is considered a mechanism for enhancing performance [41], and the results of this study provide some evidence that this is the case in both commercial and research-grade accelerometers.

Another strength of this study is the testing of numerous algorithm and device combinations. A previous study developed a multilayer neural network that was trained on a wearable system including a vest for electrocardiogram measurements and 4 accelerometers (one on each wrist and thigh) [47]. Despite the small bias, this is unlikely to be a feasible means of assessing free-living energy balance behaviors. Participant discomfort and sensor removal present additional biases (ie, missing data), which may require additional modeling approaches to address [48-50]. The threshold of practicality varies depending on the size, duration, computational resources, and specific aims of the research study. Therefore, the development of three models with varying requirements is a central advantage of this study.

Testing both classification and regression algorithms in the same devices enhances the use of the results of this study. One area of future work is to explore combined classification and regression approaches, similar to the branched models of the Actiheart [51] or stacked ensemble approaches. This may be effective in producing refined estimates of total daily energy expenditure in free-living subjects, given that most of a day comprises resting or sedentary minutes and some of our models slightly overestimate sedentary activities, although depending on the classification or regression methods, this could incur additional computational costs when applying this to larger data sets. Future work in our lab will examine the application of such models to free-living environments against a doubly labeled water criterion.

A limitation of this study is the lack of a true testing set. Rather, we attempt to develop an unbiased estimate of the true test error by (1) testing on unseen participants and (2) testing on an unseen data set. In the former, the within-subject data are generally more correlated than the between-subject data, and this method represents the closest approximation of how such a model would perform in practice [8]. In the latter, this is extended so that the training and testing sets comprised different participants and protocols. Beyond these validation approaches, the ultimate test of the results presented here is a free-living validation for energy expenditure and intensity classes. The total daily energy expenditure can be validated using the doubly labeled water method over a 7- to 14-day period [52], and the results presented in this paper are part of a wider project in which we aim to validate model predictions in free-living. Although free-living validations are critical, the resolution required to evaluate activity-specific errors can only be obtained from indirect calorimetry. Regarding activity categories, no gold standard method exists to validate time in sedentary activity, light physical activity, and MVPA outside of a controlled environment, and the generalizability of classification models to free-living studies is somewhat uncertain. The authors have highlighted the limitations of accelerometer data collected within a laboratory [53,54]; the activities performed in a free-living environment are more diverse, which further necessitates the need for more naturalistic (ie, free-living) validation studies or at least validation studies conducted over several days using diverse activity protocols in a residential facility. Next, to replicate predictions made by the present algorithms in free-living subjects, measured RMR may be required, which increases the researcher and participant burden. A suitable alternative in the absence of measured RMR would be prediction equations derived from BMI, age, height, and gender, rather than assuming a resting value of 3.5 ml O_2_/kg/min [55,56]. Finally, our use of the measured RMR to calculate *METs* may contribute to differences between the tested algorithms and the SenseWear manufacturer.

### Conclusions

This study builds on previous work from our lab and others, demonstrating that machine learning techniques can be used to learn the complexities of human movement and physiological data in the study of human energy expenditure. Classification and regression errors were greater when comparisons were made between studies. Single-sample, cross-sectional studies generating energy expenditure models show acceptable accuracy; however, it is likely that these models are overfitted to a given sample, and thus, improving generalizability is essential. To extend the utility of energy expenditure estimates beyond lab conditions, more cross testing between data sets is required, in addition to validation in free-living samples by doubly labeled water.

## Appendix

Multimedia Appendix 1 Hyperparameters used in each of the models.

Multimedia Appendix 2 Leave-one-subject-out cross-validation results for each of the regression models in each of the activity categories.

Multimedia Appendix 3 Between-model comparisons for root mean square error in each of the tested activity types.

Multimedia Appendix 4 Permutation importance analysis for Fitbit, SenseWear, and Actigraph datasets.

Multimedia Appendix 5 Leave-one-subject-out cross-validation results for each of the classification models in each of the intensity categories.

## Abbreviations

KNN: k-nearest neighbor
LOSO: leave-one-subject-out cross-validation
MAPE: mean absolute percentage error
MET: metabolic equivalent
MVPA: moderate-to-vigorous physical activity
RMR: resting metabolic rate
RMSE: root mean square error

## References

[1] Westerterp KR (2013). Physical activity and physical activity induced energy expenditure in humans: measurement, determinants, and effects. Front Physiol.

[2] Warburton DE, Nicol CW, Bredin SS (2006). Health benefits of physical activity: the evidence. Can Med Assoc J.

[3] O'Driscoll R, Turicchi J, Beaulieu K, Scott S, Matu J, Deighton K, Finlayson G, Stubbs J (2020). How well do activity monitors estimate energy expenditure? A systematic review and meta-analysis of the validity of current technologies. Br J Sports Med.

[4] Shcherbina A, Mattsson C, Waggott D, Salisbury H, Christle JW, Hastie T, Wheeler M, Ashley E (2017). Accuracy in wrist-worn, sensor-based measurements of heart rate and energy expenditure in a diverse cohort. J Pers Med.

[5] Rothney MP, Neumann M, Béziat A, Chen KY (2007). An artificial neural network model of energy expenditure using nonintegrated acceleration signals. J Appl Physiol (1985).

[6] Pober DM, Staudenmayer J, Raphael C, Freedson PS (2006). Development of novel techniques to classify physical activity mode using accelerometers. Med Sci Sports Exerc.

[7] Staudenmayer J, Pober D, Crouter S, Bassett D, Freedson P (2009). An artificial neural network to estimate physical activity energy expenditure and identify physical activity type from an accelerometer. J Appl Physiol (1985).

[8] Ellis K, Kerr J, Godbole S, Lanckriet G, Wing D, Marshall S (2014). A random forest classifier for the prediction of energy expenditure and type of physical activity from wrist and hip accelerometers. Physiol Meas.

[9] Montoye AH, Begum M, Henning Z, Pfeiffer KA (2017). Comparison of linear and non-linear models for predicting energy expenditure from raw accelerometer data. Physiol Meas.

[10] Ellis K, Kerr J, Godbole S, Staudenmayer J, Lanckriet G (2016). Hip and wrist accelerometer algorithms for free-living behavior classification. Med Sci Sports Exerc.

[11] Ahmadi MN, Chowdhury A, Pavey T, Trost SG (2020). Laboratory-based and free-living algorithms for energy expenditure estimation in preschool children: a free-living evaluation. PLoS One.

[12] Shook RP, Hand GA, O'Connor DP, Thomas DM, Hurley TG, Hébert JR, Drenowatz C, Welk GJ, Carriquiry AL, Blair SN (2018). Energy intake derived from an energy balance equation, validated activity monitors, and dual X-Ray absorptiometry can provide acceptable caloric intake data among young adults. J Nutr.

[13] Ostendorf DM, Lyden K, Pan Z, Wyatt HR, Hill JO, Melanson EL, Catenacci VA (2018). Objectively measured physical activity and sedentary behavior in successful weight loss maintainers. Obesity.

[14] Lyden K, Kozey SL, Staudenmeyer JW, Freedson PS (2011). A comprehensive evaluation of commonly used accelerometer energy expenditure and MET prediction equations. Eur J Appl Physiol.

[15] O'Driscoll R, Turicchi J, Hopkins M, Horgan GW, Finlayson G, Stubbs JR (2020). Improving energy expenditure estimates from wearable devices: a machine learning approach. J Sports Sci.

[16] Chowdhury AK, Tjondronegoro D, Chandran V, Trost SG (2017). Ensemble methods for classification of physical activities from wrist accelerometry. Med Sci Sports Exerc.

[17] Wolpert DH, Macready WG (1997). No free lunch theorems for optimization. IEEE Trans Evol Computat.

[18] Farrahi V, Niemela M, Tjurin P, Kangas M, Korpelainen R, Jamsa T (2020). Evaluating and enhancing the generalization performance of machine learning models for physical activity intensity prediction from raw acceleration data. IEEE J Biomed Health Inform.

[19] Calabró MA, Lee J, Saint-Maurice PF, Yoo H, Welk GJ (2014). Validity of physical activity monitors for assessing lower intensity activity in adults. Int J Behav Nutr Phys Act.

[20] Sanchez-Delgado G, Alcantara JM, Ortiz-Alvarez L, Xu H, Martinez-Tellez B, Labayen I, Ruiz JR (2018). Reliability of resting metabolic rate measurements in young adults: Impact of methods for data analysis. Clin Nutr.

[21] Müller MJ, Bosy-Westphal A, Klaus S, Kreymann G, Lührmann PM, Neuhäuser-Berthold M, Noack R, Pirke KM, Platte P, Selberg O, Steiniger J (2004). World health organization equations have shortcomings for predicting resting energy expenditure in persons from a modern, affluent population: generation of a new reference standard from a retrospective analysis of a German database of resting energy expenditure. Am J Clin Nutr.

[22] Byrne NM, Hills AP, Hunter GR, Weinsier RL, Schutz Y (2005). Metabolic equivalent: one size does not fit all. J Appl Physiol.

[23] Melzer K, Heydenreich J, Schutz Y, Renaud A, Kayser B, Mäder U (2016). Metabolic equivalent in adolescents, active adults and pregnant women. Nutrients.

[24] Brage S, Brage N, Franks PW, Ekelund U, Wareham NJ (2005). Reliability and validity of the combined heart rate and movement sensor Actiheart. Eur J Clin Nutr.

[25] Whybrow S, Ritz P, Horgan G, Stubbs R (2012). An evaluation of the IDEEA™ activity monitor for estimating energy expenditure. Br J Nutr.

[26] Ceesay SM, Prentice AM, Day KC, Murgatroyd PR, Goldberg GR, Scott W, Spurr GB (1989). The use of heart rate monitoring in the estimation of energy expenditure: a validation study using indirect whole-body calorimetry. Br J Nutr.

[27] McArdle WD, Katch FI, Katch VL (2010). Exercise Physiology: Nutrition, Energy, and Human Performance.

[28] Blair CK, Morey MC, Desmond RA, Cohen HJ, Sloane R, Snyder DC, Demark-Wahnefried W (2014). Light-intensity activity attenuates functional decline in older cancer survivors. Med Sci Sports Exerc.

[29] Beaulieu K, Hopkins M, Blundell J, Finlayson G (2017). Impact of physical activity level and dietary fat content on passive overconsumption of energy in non-obese adults. Int J Behav Nutr Phys Act.

[30] Géron A (2019). Hands-On Machine Learning with Scikit-Learn, Keras, and TensorFlow: Concepts, Tools, and Techniques to Build Intelligent Systems 2nd Edition.

[31] Pedregosa F, Weiss R, Brucher M, Varoquaux G, Gramfort A, Michel V (2011). Scikit-learn: machine learning in python. J Mach Learn Res.

[32] Chollet F (2015). Keras-team/keras: Deep Learning for Humans. GitHub.

[33] Breiman L (2001). Random Forests. Mach Learn.

[34] Hastie T, Tibshirani R, Friedman J (2009). The Elements of Statistical Learning: Data Mining, Inference, and Prediction, Second Edition.

[35] Kuhn M, Johnson K (2013). Applied Predictive Modeling.

[36] Bates D, Mächler M, Bolker B, Walker S (2015). Fitting linear mixed-effects models using. J Stat Soft.

[37] Kuhn M (2008). Building predictive models in R using the caret package. J Stat Soft.

[38] Kate RJ, Swartz AM, Welch WA, Strath SJ (2016). Comparative evaluation of features and techniques for identifying activity type and estimating energy cost from accelerometer data. Physiol Meas.

[39] DeGregory KW, Kuiper P, DeSilvio T, Pleuss JD, Miller R, Roginski JW, Fisher CB, Harness D, Viswanath S, Heymsfield SB, Dungan I, Thomas DM (2018). A review of machine learning in obesity. Obes Rev.

[40] Paraschiakos S, de Sá SC, Okai J, Slagboom EP, Beekman M, Knobbe A (2020). RNNs on Monitoring Physical Activity Energy Expenditure in Older People. arXiv.

[41] Vabalas A, Gowen E, Poliakoff E, Casson AJ (2019). Machine learning algorithm validation with a limited sample size. PLoS One.

[42] Willetts M, Hollowell S, Aslett L, Holmes C, Doherty A (2018). Statistical machine learning of sleep and physical activity phenotypes from sensor data in 96,220 UK Biobank participants. Sci Rep.

[43] Sasaki JE, Hickey AM, Staudenmayer JW, John D, Kent JA, Freedson PS (2016). Performance of activity classification algorithms in free-living older adults. Med Sci Sports Exerc.

[44] Montoye AH, Westgate BS, Fonley MR, Pfeiffer KA (2018). Cross-validation and out-of-sample testing of physical activity intensity predictions with a wrist-worn accelerometer. J Appl Physiol.

[45] Feehan LM, Geldman J, Sayre EC, Park C, Ezzat AM, Yoo JY, Hamilton CB, Li LC (2018). Accuracy of fitbit devices: systematic review and narrative syntheses of quantitative data. JMIR Mhealth Uhealth.

[46] Zhang S, Rowlands AV, Murray P, Hurst TL (2012). Physical activity classification using the GENEA wrist-worn accelerometer. Med Sci Sports Exerc.

[47] Lu K, Yang L, Seoane F, Abtahi F, Forsman M, Lindecrantz K (2018). Fusion of heart rate, respiration and motion measurements from a wearable sensor system to enhance energy expenditure estimation. Sensors.

[48] Lee P (2013). Data imputation for accelerometer-measured physical activity: the combined approach. Am J Clin Nutr.

[49] Xu SY, Nelson S, Kerr J, Godbole S, Patterson R, Merchant G, Abramson I, Staudenmayer J, Natarajan L (2018). Statistical approaches to account for missing values in accelerometer data: applications to modeling physical activity. Stat Methods Med Res.

[50] O'Driscoll R, Turicchi J, Duarte C, Michalowska J, Larsen SC, Palmeira AL, Heitmann BL, Horgan GW, Stubbs RJ (2020). A novel scaling methodology to reduce the biases associated with missing data from commercial activity monitors. PLoS One.

[51] Brage S, Brage N, Franks PW, Ekelund U, Wong M, Andersen LB, Froberg K, Wareham NJ (2004). Branched equation modeling of simultaneous accelerometry and heart rate monitoring improves estimate of directly measured physical activity energy expenditure. J Appl Physiol.

[52] Black AE, Cole TJ (2000). Within- and between-subject variation in energy expenditure measured by the doubly-labelled water technique: implications for validating reported dietary energy intake. Eur J Clin Nutr.

[53] Bastian T, Maire A, Dugas J, Ataya A, Villars C, Gris F, Perrin E, Caritu Y, Doron M, Blanc S, Jallon P, Simon C (2015). Automatic identification of physical activity types and sedentary behaviors from triaxial accelerometer: laboratory-based calibrations are not enough. J Appl Physiol.

[54] Kerr J, Marshall SJ, Godbole S, Chen J, Legge A, Doherty AR, Kelly P, Oliver M, Badland HM, Foster C (2013). Using the SenseCam to improve classifications of sedentary behavior in free-living settings. Am J Prev Med.

[55] Kim D, Lee J, Park HK, Jang DP, Song S, Cho BH, Jung Y, Park R, Joo N, Kim IY (2017). Comparing the standards of one metabolic equivalent of task in accurately estimating physical activity energy expenditure based on acceleration. J Sports Sci.

[56] Kozey SL, Lyden K, Howe CA, Staudenmayer JW, Freedson PS (2010). Accelerometer output and MET values of common physical activities. Med Sci Sports Exerc.

