# Supplementary material for: Comparison of the Validity and Generalizability of Machine Learning Algorithms for the Prediction of Energy Expenditure: Validation Study
Source: JMIR Mhealth Uhealth. 2021 Aug 4;9(8):e23938. doi: 10.2196/23938 (PMC8374660; doi:10.2196/23938)
Supplement: Multimedia Appendix 1 [file mhealth_v9i8e23938_app1.docx]

**Multimedia Appendix 1.** Hyperparameters used in each of the models.

**Fitbit Regression models**

**Study 1 as training**

**Random Forest**

{'n_estimators': 600,

'min_samples_split': 2,

'min_samples_leaf': 4,

'max_features': 'sqrt',

'max_depth': 60,

'bootstrap': True}

**Gradient boost**

{'n_estimators': 1000,

'min_samples_split': 6,

'min_samples_leaf': 2,

'max_features': 'log2',

'max_depth': 20,

'loss': 'ls',

'learning_rate': 0.01}

**Neural network**

{'n_neurons': 65,

'n_hidden': 2,

'learning_rate': 0.0001}

**Study 2 as training**

**Random forest**

{'n_estimators': 200,

'min_samples_split': 2,

'min_samples_leaf': 2,

'max_features': 'sqrt',

'max_depth': 30,

'bootstrap': True}

**Gradient boost**

{'n_estimators': 1000,

'min_samples_split': 4,

'min_samples_leaf': 8,

'max_features': 'auto',

'max_depth': 2,

'loss': 'ls',

'learning_rate': 0.01}

**Neural network**

{'n_neurons': 15,

'n_hidden': 3,

'learning_rate': 0.0001}

**LOSO**

Random forest

{'n_estimators': 800,

'min_samples_split': 10,

'min_samples_leaf': 1,

'max_features': 'sqrt',

'max_depth': 60,

'bootstrap': True}

**Gradient boost**

{'n_estimators': 1000,

'min_samples_split': 10,

'min_samples_leaf': 1,

'max_features': 'sqrt',

'max_depth': 15,

'loss': 'ls',

'learning_rate': 0.01}

**Neural network**

{'n_neurons': 65,

'n_hidden': 2,

'learning_rate': 0.0001}

**Sensewear regression models**

**Study 1 as training**

Random forest

{ 'n_estimators': 200,

'min_samples_split': 5,

'min_samples_leaf': 4,

'max_features': 'sqrt',

'max_depth': 40,

'bootstrap': True}

**Gradient boost**

{ 'n_estimators': 10000,

'min_samples_split': 10,

'min_samples_leaf': 1,

'max_features': 'log2',

'max_depth': 15,

'learning_rate': 0.01}

**Neural network**

{'n_neurons': 15,

'n_hidden': 3,

'learning_rate': 0.0001}

**Study 2 as training**

**Random forest**

{‘n_estimators': 800,

'min_samples_split': 10,

'min_samples_leaf': 1,

'max_features': 'sqrt',

'max_depth': 60,

'bootstrap': True}

**Gradient boost**

{'n_estimators': 1000,

'min_samples_split': 10,

'min_samples_leaf': 1,

'max_features': 'sqrt',

'max_depth': 15,

'learning_rate': 0.01}

**Neural network**

{'n_neurons': 65,

'n_hidden': 2,

'learning_rate': 0.0001}

**LOSO**

**Random forest**

{'n_estimators': 800,

'min_samples_split': 5,

'min_samples_leaf': 1,

'max_features': 'sqrt',

'max_depth': 30,

'bootstrap': True}

**Gradient boost**

{'n_estimators': 10000,

'min_samples_split': 6,

'min_samples_leaf': 12,

'max_features': 'log2',

'max_depth': 15,

'learning_rate': 0.01}

**Neural network**

{'n_neurons': 15,

'n_hidden': 3,

'learning_rate': 0.0001}

**ActiGraph Regression models**

**Study 1 as training**

**Random Forest**

{'n_estimators': 1000,

'min_samples_split': 5,

'min_samples_leaf': 2,

'max_features': 'sqrt',

'max_depth': 50,

'bootstrap': False}

**Gradient Boost**

{'n_estimators': 10000,

'min_samples_split': 10,

'min_samples_leaf': 12,

'max_features': 'sqrt',

'max_depth': 10,

'loss': 'ls',

'learning_rate': 0.01}

**Neural Network**

{'n_neurons': 55,

'n_hidden': 3,

'learning_rate': 0.003}

**Study 2 as training**

Random forest

{'n_estimators': 200,

'min_samples_split': 2,

'min_samples_leaf': 1,

'max_features': 'sqrt',

'max_depth': 60,

'bootstrap': True}

**Gradient boosting**

{'n_estimators': 10000,

'min_samples_split': 20,

'min_samples_leaf': 2,

'max_features': 'sqrt',

'max_depth': 20,

'learning_rate': 0.01}

**Neural network**

{ 'n_neurons': 65, 'n_hidden': 2, 'learning_rate': 0.0001}

**LOSO**

Random forest

{'n_estimators': 1000,

'min_samples_split': 5,

'min_samples_leaf': 1,

'max_features': 'sqrt',

'max_depth': 60,

'bootstrap': False}

**Gradient Boost**

{'n_estimators': 10000,

'min_samples_split': 6,

'min_samples_leaf': 12,

'max_features': 'log2',

'max_depth': 15,

'learning_rate': 0.01}

**Neural Network**

{ 'n_neurons': 55,

'n_hidden': 2,

'learning_rate': 0.003

}

**Classification models**

**Fitbit classification models**

**Study 1 as training**

**KNN**

{'weights': 'uniform',

'n_neighbors': 15,

'metric': 'minkowski'}

**Random forest**

{'n_estimators': 1000,

'min_samples_split': 10,

'min_samples_leaf': 4,

'max_features': 'auto',

'max_depth': 80,

'bootstrap': True}

**Gradient boost**

{'n_estimators': 1000,

'min_samples_split': 2,

'min_samples_leaf': 1,

'max_features': 'log2',

'max_depth': 15,

'learning_rate': 0.1}

**Neural network**

{ 'n_neurons': 65,

'n_hidden': 2,

'learning_rate': 0.0001}

**SVM**

{ 'C': 5.046137691733707,

'gamma': 0.19767211400638388}

**Study 2 as training**

**KNN**

{'weights': 'uniform',

'n_neighbors': 33,

'metric': 'manhattan'}

**Random forest**

{'n_estimators': 800,

'min_samples_split': 5,

'min_samples_leaf': 4,

'max_features': 'auto',

'max_depth': 80,

'bootstrap': True}

**Gradient boost**

{'n_estimators': 1000,

'min_samples_split': 6,

'min_samples_leaf': 2,

'max_features': 'sqrt',

'max_depth': 2,

'learning_rate': 0.01}

**Neural network**

{ 'n_neurons': 15, 'n_hidden': 3, 'learning_rate': 0.0001}

**SVM**

{ 'C': 7.924145688620425, 'gamma': 0.14645041271999773}

**LOSO**

**KNN**

{ 'weights': 'distance', 'n_neighbors': 73, 'metric': 'manhattan'}

**Random forest**

{ 'n_estimators': 400,

'min_samples_split': 5,

'min_samples_leaf': 4,

'max_features': 'sqrt',

'max_depth': 70,

'bootstrap': True}

**Gradient boost**

{ 'n_estimators': 1000,

'min_samples_split': 4,

'min_samples_leaf': 8,

'max_features': 'auto',

'max_depth': 2,

'learning_rate': 0.01}

**Neural network**

{ 'n_neurons': 70, 'n_hidden': 2, 'learning_rate': 0.1}

**SVM**

{'C': 6.319450186421157, 'gamma': 0.3912291401980419}

**Sensewear classification models**

**Study 1 as training**

**KNN**

{'weights': 'uniform',

'n_neighbors': 27,

'metric': 'manhattan'}

**Random Forest**

{'n_estimators': 800,

'min_samples_split': 10,

'min_samples_leaf': 4,

'max_features': 'auto',

'max_depth': 40,

'bootstrap': False}

**Gradient boost**

{'n_estimators': 1000,

'min_samples_split': 4,

'min_samples_leaf': 4,

'max_features': 'log2',

'max_depth': 10,

'learning_rate': 0.1}

**Neural network**

{ 'n_neurons': 15, 'n_hidden': 3, 'learning_rate': 0.0001}

**SVM**

{ 'C': 7.924145688620425, 'gamma': 0.14645041271999773}

**Study 2 as training**

**KNN**

{'weights': 'uniform', 'n_neighbors': 53, 'metric': 'manhattan'}

**Random forest**

{ 'n_estimators': 1000,

'min_samples_split': 5,

'min_samples_leaf': 4,

'max_features': 'sqrt',

'max_depth': 40,

'bootstrap': True}

**Gradient boost**

{ 'n_estimators': 10000,

'min_samples_split': 6,

'min_samples_leaf': 12,

'max_features': 'sqrt',

'max_depth': 20,

'learning_rate': 0.1

}

**Neural network**

{ 'n_neurons': 15, 'n_hidden': 3, 'learning_rate': 0.0001}

**SVM**

{ 'C': 7.924145688620425, 'gamma': 0.14645041271999773}

**LOSO**

**KNN**

{'weights': 'uniform',

'n_neighbors': 33,

'metric': 'manhattan'}

**Random forest**

'n_estimators': 400,

'min_samples_split': 5,

'min_samples_leaf': 4,

'max_features': 'sqrt',

'max_depth': 70,

'bootstrap': True

}

**Gradient boost**

{ 'n_estimators': 1000,

'min_samples_split': 4,

'min_samples_leaf': 2,

'max_features': 'log2',

'max_depth': 10,

'learning_rate': 0.05

}

**Neural network**

{'n_neurons': 65,

'n_hidden': 2,

'learning_rate': 0.0001

}

**SVM**

{ 'C': 7.924145688620425,

'gamma': 0.14645041271999773}

**ActiGraph classification models**

**Study 1 as training**

**KNN**

{'weights': 'uniform',

'n_neighbors': 41,

'metric': 'manhattan'}

**Random forest**

{'n_estimators': 200,

'min_samples_split': 2,

'min_samples_leaf': 1,

'max_features': 'sqrt',

'max_depth': 90,

'bootstrap': False}

**Gradient boost**

{'n_estimators': 1000,

'min_samples_split': 20,

'min_samples_leaf': 4,

'max_features': 'sqrt',

'max_depth': 10,

'learning_rate': 0.1}

**Neural network**

{ 'n_neurons': 65,

'n_hidden': 2,

'learning_rate': 0.0001}

**SVM**

{ 'C': 7.924145688620425,

'gamma': 0.14645041271999773}

**Study 2 as training**

**KNN**

{ 'weights': 'uniform', 'n_neighbors': 17, 'metric': 'manhattan'}

**Random forest**

{ 'n_estimators': 800,

'min_samples_split': 5,

'min_samples_leaf': 4,

'max_features': 'auto',

'max_depth': 80,

'bootstrap': True

}

**Gradient boost**

{ 'n_estimators': 1000,

'min_samples_split': 6,

'min_samples_leaf': 8,

'max_features': 'log2',

'max_depth': 20,

'learning_rate': 0.01

}

**Neural network**

{ 'n_neurons': 15, 'n_hidden': 3, 'learning_rate': 0.0001}

**SVM**

{ 'C': 7.924145688620425,

'gamma': 0.14645041271999773

}

**LOSO**

**KNN**

{'weights': 'distance',

'n_neighbors': 73,

'metric': 'manhattan'}

**Random Forest**

{'n_estimators': 1000,

'min_samples_split': 10,

'min_samples_leaf': 4,

'max_features': 'auto',

'max_depth': 20,

'bootstrap': True}

**Gradient boost**

{ 'n_estimators': 1000,

'min_samples_split': 6,

'min_samples_leaf': 2,

'max_features': 'log2',

'max_depth': 3,

'learning_rate': 0.01

}

**Neural network**

{ 'n_neurons': 65,

'n_hidden': 2,

'learning_rate': 0.0001}

**SVM**

{ 'C': 7.924145688620425,

'gamma': 0.14645041271999773}
