# Supplementary material for: Comparison of the Validity and Generalizability of Machine Learning Algorithms for the Prediction of Energy Expenditure: Validation Study
Source: JMIR Mhealth Uhealth. 2021 Aug 4;9(8):e23938. doi: 10.2196/23938 (PMC8374660; doi:10.2196/23938)
Supplement: Multimedia Appendix 2 [file mhealth_v9i8e23938_app2.docx]

**Multimedia Appendix 2.** Leave-one-subject-out cross-validation results for each of the regression models in each of the activity categories.

| **Model** | **Activity** | **Predicted (METs)** | **True (METs)** | **MAPE** | **RMSE** | **CCC** |
| --- | --- | --- | --- | --- | --- | --- |
| AG Gradient Boost | ADL | 2.82 ± 0.91 | 2.56 ± 0.89 | 22.93 | 0.72 | 0.69 (0.66, 0.72) |
| AG Gradient Boost | Cycling | 4.7 ± 1.29 | 4.82 ± 1.59 | 16.54 | 1.07 | 0.73 (0.7, 0.76) |
| AG Gradient Boost | Elliptical | 6.75 ± 1.52 | 7.04 ± 2.13 | 15.06 | 1.52 | 0.67 (0.61, 0.72) |
| AG Gradient Boost | Rowing | 6.35 ± 1.55 | 6.51 ± 2.04 | 14.34 | 1.24 | 0.76 (0.72, 0.8) |
| AG Gradient Boost | Running | 8.25 ± 1.3 | 8.52 ± 1.66 | 13.55 | 1.41 | 0.56 (0.5, 0.61) |
| AG Gradient Boost | Sedentary | 1.37 ± 0.34 | 1.3 ± 0.34 | 19.84 | 0.37 | 0.4 (0.35, 0.45) |
| AG Gradient Boost | Transitional | 3.06 ± 1.83 | 2.99 ± 1.99 | 19.96 | 0.77 | 0.92 (0.91, 0.93) |
| AG Gradient Boost | Walking | 4.2 ± 0.75 | 4.22 ± 0.99 | 14.46 | 0.78 | 0.61 (0.57, 0.65) |
| AG Neural Network | ADL | 2.69 ± 1.07 | 2.56 ± 0.89 | 26.10 | 0.88 | 0.61 (0.57, 0.64) |
| AG Neural Network | Cycling | 4.75 ± 1.64 | 4.82 ± 1.59 | 20.72 | 1.29 | 0.68 (0.64, 0.71) |
| AG Neural Network | Elliptical | 6.9 ± 1.81 | 7.04 ± 2.13 | 18.08 | 1.62 | 0.66 (0.6, 0.72) |
| AG Neural Network | Rowing | 6.26 ± 1.9 | 6.51 ± 2.04 | 16.74 | 1.39 | 0.75 (0.7, 0.8) |
| AG Neural Network | Running | 8.41 ± 1.82 | 8.52 ± 1.66 | 16.72 | 1.82 | 0.45 (0.38, 0.52) |
| AG Neural Network | Sedentary | 1.34 ± 0.41 | 1.3 ± 0.34 | 24.36 | 0.46 | 0.25 (0.19, 0.3) |
| AG Neural Network | Transitional | 3.1 ± 2.09 | 2.99 ± 1.99 | 25.22 | 1.06 | 0.86 (0.84, 0.88) |
| AG Neural Network | Walking | 4.26 ± 1.12 | 4.22 ± 0.99 | 17.71 | 0.97 | 0.57 (0.53, 0.62) |
| AG Random Forest | ADL | 2.86 ± 0.92 | 2.56 ± 0.89 | 24.16 | 0.74 | 0.68 (0.65, 0.71) |
| AG Random Forest | Cycling | 4.71 ± 1.24 | 4.82 ± 1.59 | 16.72 | 1.06 | 0.72 (0.69, 0.75) |
| AG Random Forest | Elliptical | 6.76 ± 1.47 | 7.04 ± 2.13 | 15.15 | 1.52 | 0.66 (0.6, 0.71) |
| AG Random Forest | Rowing | 6.3 ± 1.49 | 6.51 ± 2.04 | 14.31 | 1.25 | 0.75 (0.71, 0.79) |
| AG Random Forest | Running | 8.22 ± 1.24 | 8.52 ± 1.66 | 13.59 | 1.42 | 0.54 (0.48, 0.59) |
| AG Random Forest | Sedentary | 1.38 ± 0.35 | 1.3 ± 0.34 | 20.31 | 0.39 | 0.38 (0.33, 0.43) |
| AG Random Forest | Transitional | 3.09 ± 1.79 | 2.99 ± 1.99 | 21.01 | 0.79 | 0.91 (0.9, 0.92) |
| AG Random Forest | Walking | 4.2 ± 0.68 | 4.22 ± 0.99 | 14.54 | 0.77 | 0.59 (0.55, 0.63) |
| FB Gradient Boost | ADL | 3.27 ± 1.1 | 2.55 ± 0.89 | 41.94 | 1.22 | 0.41 (0.37, 0.45) |
| FB Gradient Boost | Cycling | 3.97 ± 1.29 | 4.76 ± 1.58 | 25.30 | 1.67 | 0.42 (0.37, 0.46) |
| FB Gradient Boost | Elliptical | 6.34 ± 1.51 | 7.01 ± 2.16 | 21.49 | 1.95 | 0.48 (0.4, 0.56) |
| FB Gradient Boost | Rowing | 4.95 ± 1.24 | 6.49 ± 2.05 | 25.57 | 2.39 | 0.3 (0.23, 0.36) |
| FB Gradient Boost | Running | 8.3 ± 1.31 | 8.51 ± 1.67 | 14.46 | 1.50 | 0.5 (0.44, 0.56) |
| FB Gradient Boost | Sedentary | 1.55 ± 0.53 | 1.29 ± 0.34 | 34.74 | 0.65 | 0.08 (0.03, 0.13) |
| FB Gradient Boost | Transitional | 3.47 ± 1.61 | 2.98 ± 1.99 | 46.04 | 1.29 | 0.76 (0.72, 0.79) |
| FB Gradient Boost | Walking | 4.52 ± 0.97 | 4.21 ± 0.99 | 19.01 | 1.00 | 0.51 (0.46, 0.55) |
| FB Neural Network | ADL | 3.21 ± 1.27 | 2.55 ± 0.89 | 43.44 | 1.34 | 0.37 (0.33, 0.41) |
| FB Neural Network | Cycling | 3.89 ± 1.41 | 4.76 ± 1.58 | 27.88 | 1.81 | 0.38 (0.33, 0.43) |
| FB Neural Network | Elliptical | 6.32 ± 1.81 | 7.01 ± 2.16 | 24.55 | 2.08 | 0.49 (0.4, 0.56) |
| FB Neural Network | Rowing | 5.23 ± 1.43 | 6.49 ± 2.05 | 24.89 | 2.26 | 0.35 (0.27, 0.43) |
| FB Neural Network | Running | 8.31 ± 1.52 | 8.51 ± 1.67 | 15.94 | 1.64 | 0.48 (0.41, 0.54) |
| FB Neural Network | Sedentary | 1.53 ± 0.52 | 1.29 ± 0.34 | 37.52 | 0.66 | 0.01 (-0.03, 0.06) |
| FB Neural Network | Transitional | 3.42 ± 1.73 | 2.98 ± 1.99 | 48.18 | 1.41 | 0.72 (0.68, 0.76) |
| FB Neural Network | Walking | 4.55 ± 1.1 | 4.21 ± 0.99 | 21.17 | 1.08 | 0.49 (0.44, 0.54) |
| FB Random Forest | ADL | 3.31 ± 1.1 | 2.55 ± 0.89 | 42.51 | 1.23 | 0.41 (0.37, 0.45) |
| FB Random Forest | Cycling | 3.95 ± 1.25 | 4.76 ± 1.58 | 24.50 | 1.66 | 0.42 (0.37, 0.46) |
| FB Random Forest | Elliptical | 6.29 ± 1.38 | 7.01 ± 2.16 | 20.53 | 1.92 | 0.48 (0.4, 0.55) |
| FB Random Forest | Rowing | 4.91 ± 1.13 | 6.49 ± 2.05 | 25.31 | 2.40 | 0.28 (0.21, 0.34) |
| FB Random Forest | Running | 8.21 ± 1.24 | 8.51 ± 1.67 | 14.33 | 1.49 | 0.5 (0.43, 0.55) |
| FB Random Forest | Sedentary | 1.54 ± 0.49 | 1.29 ± 0.34 | 33.73 | 0.62 | 0.08 (0.03, 0.13) |
| FB Random Forest | Transitional | 3.49 ± 1.56 | 2.98 ± 1.99 | 47.27 | 1.29 | 0.75 (0.71, 0.78) |
| FB Random Forest | Walking | 4.54 ± 0.95 | 4.21 ± 0.99 | 19.18 | 0.99 | 0.51 (0.46, 0.55) |
| SWA Gradient Boost | ADL | 2.85 ± 1.04 | 2.56 ± 0.89 | 23.27 | 0.82 | 0.66 (0.62, 0.69) |
| SWA Gradient Boost | Cycling | 4.66 ± 1.34 | 4.84 ± 1.58 | 16.60 | 1.07 | 0.74 (0.71, 0.77) |
| SWA Gradient Boost | Elliptical | 6.97 ± 1.7 | 7.13 ± 2.12 | 14.16 | 1.39 | 0.74 (0.69, 0.78) |
| SWA Gradient Boost | Rowing | 6.08 ± 1.67 | 6.58 ± 2.04 | 14.42 | 1.29 | 0.77 (0.72, 0.81) |
| SWA Gradient Boost | Running | 8.31 ± 1.35 | 8.54 ± 1.66 | 12.00 | 1.25 | 0.66 (0.61, 0.7) |
| SWA Gradient Boost | Sedentary | 1.36 ± 0.35 | 1.3 ± 0.34 | 20.34 | 0.42 | 0.29 (0.23, 0.34) |
| SWA Gradient Boost | Transitional | 3.14 ± 1.88 | 3 ± 1.99 | 21.87 | 0.82 | 0.91 (0.89, 0.92) |
| SWA Gradient Boost | Walking | 4.26 ± 0.88 | 4.24 ± 0.99 | 12.89 | 0.69 | 0.73 (0.69, 0.76) |
| SWA Manufacturer | ADL | 3.73 ± 2.05 | 2.57 ± 0.89 | 66.52 | 2.28 | 0.18 (0.14, 0.21) |
| SWA Manufacturer | Cycling | 3.31 ± 1.88 | 4.83 ± 1.58 | 38.76 | 2.18 | 0.43 (0.39, 0.47) |
| SWA Manufacturer | Elliptical | 6 ± 1.57 | 7.13 ± 2.12 | 24.41 | 2.67 | 0.13 (0.04, 0.22) |
| SWA Manufacturer | Rowing | 6.14 ± 2.1 | 6.58 ± 2.04 | 33.96 | 2.68 | 0.18 (0.06, 0.28) |
| SWA Manufacturer | Running | 8.11 ± 1.69 | 8.54 ± 1.66 | 21.95 | 2.28 | 0.1 (0.02, 0.18) |
| SWA Manufacturer | Sedentary | 1.22 ± 0.27 | 1.3 ± 0.34 | 21.44 | 0.40 | 0.15 (0.09, 0.21) |
| SWA Manufacturer | Transitional | 2.77 ± 1.79 | 3 ± 1.99 | 31.17 | 1.46 | 0.7 (0.66, 0.74) |
| SWA Manufacturer | Walking | 3.92 ± 0.85 | 4.24 ± 0.99 | 21.64 | 1.19 | 0.21 (0.15, 0.27) |
| SWA Neural Network | ADL | 2.78 ± 1.07 | 2.56 ± 0.89 | 24.26 | 0.82 | 0.66 (0.63, 0.69) |
| SWA Neural Network | Cycling | 4.65 ± 1.44 | 4.84 ± 1.58 | 17.30 | 1.14 | 0.72 (0.69, 0.75) |
| SWA Neural Network | Elliptical | 7.05 ± 1.8 | 7.13 ± 2.12 | 15.45 | 1.46 | 0.72 (0.66, 0.77) |
| SWA Neural Network | Rowing | 6.25 ± 1.83 | 6.58 ± 2.04 | 15.77 | 1.30 | 0.78 (0.73, 0.82) |
| SWA Neural Network | Running | 8.39 ± 1.51 | 8.54 ± 1.66 | 11.77 | 1.26 | 0.68 (0.64, 0.73) |
| SWA Neural Network | Sedentary | 1.36 ± 0.44 | 1.3 ± 0.34 | 24.07 | 0.51 | 0.16 (0.1, 0.22) |
| SWA Neural Network | Transitional | 3.13 ± 2.02 | 3 ± 1.99 | 25.22 | 0.91 | 0.9 (0.88, 0.91) |
| SWA Neural Network | Walking | 4.26 ± 0.99 | 4.24 ± 0.99 | 14.55 | 0.76 | 0.71 (0.67, 0.74) |
| SWA Random Forest | ADL | 2.93 ± 1.06 | 2.56 ± 0.89 | 24.88 | 0.86 | 0.63 (0.6, 0.67) |
| SWA Random Forest | Cycling | 4.66 ± 1.29 | 4.84 ± 1.58 | 16.06 | 1.05 | 0.74 (0.71, 0.77) |
| SWA Random Forest | Elliptical | 6.91 ± 1.61 | 7.13 ± 2.12 | 14.16 | 1.38 | 0.73 (0.68, 0.77) |
| SWA Random Forest | Rowing | 6.02 ± 1.64 | 6.58 ± 2.04 | 14.40 | 1.31 | 0.76 (0.71, 0.8) |
| SWA Random Forest | Running | 8.28 ± 1.26 | 8.54 ± 1.66 | 12.28 | 1.26 | 0.64 (0.59, 0.68) |
| SWA Random Forest | Sedentary | 1.37 ± 0.36 | 1.3 ± 0.34 | 20.66 | 0.43 | 0.26 (0.21, 0.32) |
| SWA Random Forest | Transitional | 3.16 ± 1.83 | 3 ± 1.99 | 22.98 | 0.82 | 0.91 (0.89, 0.92) |
| SWA Random Forest | Walking | 4.26 ± 0.83 | 4.24 ± 0.99 | 12.87 | 0.68 | 0.72 (0.69, 0.75) |

LOSO results for each of the regression models in each of the activity categories. Abbreviations: ActiGraph (AG), Fitbit (FB), SenseWear (SWA). Root mean squared error (RMSE), Mean absolute percentage error (MAPE), concordance correlation coefficient (CCC), activities of daily living (ADL).
