# Supplementary material for: Comparison of the Validity and Generalizability of Machine Learning Algorithms for the Prediction of Energy Expenditure: Validation Study
Source: JMIR Mhealth Uhealth. 2021 Aug 4;9(8):e23938. doi: 10.2196/23938 (PMC8374660; doi:10.2196/23938)
Supplement: Multimedia Appendix 3 [file mhealth_v9i8e23938_app3.docx]

**Multimedia Appendix 3**. Between-model comparisons for root mean square error in each of the tested activity types.

Overall

The model was significant (F = 100.59, p < 2.2e-16).

| contrast | estimate | SE | df | t.ratio | p.value | comparison |
| --- | --- | --- | --- | --- | --- | --- |
| AG Gradient Boost - AG Neural Network | -0.20 | 0.04 | 770.01 | -4.84 | 7.07E-05 | all |
| AG Gradient Boost - AG Random Forest | -0.01 | 0.04 | 770.01 | -0.28 | 1 | all |
| AG Gradient Boost - FB Gradient Boost | -0.37 | 0.04 | 770.84 | -8.88 | 2.08E-16 | all |
| AG Gradient Boost - FB Neural Network | -0.45 | 0.04 | 770.84 | -10.75 | 1.48E-23 | all |
| AG Gradient Boost - FB Random Forest | -0.37 | 0.04 | 770.84 | -8.81 | 3.78E-16 | all |
| AG Gradient Boost - SWA Gradient Boost | 0.03 | 0.04 | 770.30 | 0.67 | 1 | all |
| AG Gradient Boost - SWA Manufacturer | -0.88 | 0.04 | 770.61 | -21.16 | 5.05E-77 | all |
| AG Gradient Boost - SWA Neural Network | -0.01 | 0.04 | 770.30 | -0.26 | 1 | all |
| AG Gradient Boost - SWA Random Forest | 0.02 | 0.04 | 770.30 | 0.38 | 1 | all |
| AG Neural Network - AG Random Forest | 0.19 | 0.04 | 770.01 | 4.56 | 0.000264 | all |
| AG Neural Network - FB Gradient Boost | -0.17 | 0.04 | 770.84 | -4.06 | 0.002384 | all |
| AG Neural Network - FB Neural Network | -0.25 | 0.04 | 770.84 | -5.93 | 2.01E-07 | all |
| AG Neural Network - FB Random Forest | -0.17 | 0.04 | 770.84 | -3.99 | 0.003236 | all |
| AG Neural Network - SWA Gradient Boost | 0.23 | 0.04 | 770.30 | 5.51 | 2.26E-06 | all |
| AG Neural Network - SWA Manufacturer | -0.68 | 0.04 | 770.61 | -16.31 | 6.17E-50 | all |
| AG Neural Network - SWA Neural Network | 0.19 | 0.04 | 770.30 | 4.58 | 0.000249 | all |
| AG Neural Network - SWA Random Forest | 0.22 | 0.04 | 770.30 | 5.21 | 1.07E-05 | all |
| AG Random Forest - FB Gradient Boost | -0.36 | 0.04 | 770.84 | -8.61 | 1.91E-15 | all |
| AG Random Forest - FB Neural Network | -0.44 | 0.04 | 770.84 | -10.47 | 1.93E-22 | all |
| AG Random Forest - FB Random Forest | -0.36 | 0.04 | 770.84 | -8.53 | 3.41E-15 | all |
| AG Random Forest - SWA Gradient Boost | 0.04 | 0.04 | 770.30 | 0.95 | 1 | all |
| AG Random Forest - SWA Manufacturer | -0.87 | 0.04 | 770.61 | -20.88 | 2.05E-75 | all |
| AG Random Forest - SWA Neural Network | 0.00 | 0.04 | 770.30 | 0.02 | 1 | all |
| AG Random Forest - SWA Random Forest | 0.03 | 0.04 | 770.30 | 0.65 | 1 | all |
| FB Gradient Boost - FB Neural Network | -0.08 | 0.04 | 770.01 | -1.87 | 1 | all |
| FB Gradient Boost - FB Random Forest | 0.00 | 0.04 | 770.01 | 0.07 | 1 | all |
| FB Gradient Boost - SWA Gradient Boost | 0.40 | 0.04 | 770.88 | 9.55 | 7.74E-19 | all |
| FB Gradient Boost - SWA Manufacturer | -0.51 | 0.04 | 770.54 | -12.18 | 1.11E-29 | all |
| FB Gradient Boost - SWA Neural Network | 0.36 | 0.04 | 770.88 | 8.62 | 1.68E-15 | all |
| FB Gradient Boost - SWA Random Forest | 0.39 | 0.04 | 770.88 | 9.26 | 9.21E-18 | all |
| FB Neural Network - FB Random Forest | 0.08 | 0.04 | 770.01 | 1.94 | 1 | all |
| FB Neural Network - SWA Gradient Boost | 0.48 | 0.04 | 770.88 | 11.42 | 2.41E-26 | all |
| FB Neural Network - SWA Manufacturer | -0.43 | 0.04 | 770.54 | -10.30 | 9.24E-22 | all |
| FB Neural Network - SWA Neural Network | 0.44 | 0.04 | 770.88 | 10.49 | 1.67E-22 | all |
| FB Neural Network - SWA Random Forest | 0.47 | 0.04 | 770.88 | 11.13 | 4.09E-25 | all |
| FB Random Forest - SWA Gradient Boost | 0.40 | 0.04 | 770.88 | 9.47 | 1.46E-18 | all |
| FB Random Forest - SWA Manufacturer | -0.51 | 0.04 | 770.54 | -12.25 | 5.18E-30 | all |
| FB Random Forest - SWA Neural Network | 0.36 | 0.04 | 770.88 | 8.55 | 3.02E-15 | all |
| FB Random Forest - SWA Random Forest | 0.39 | 0.04 | 770.88 | 9.18 | 1.71E-17 | all |
| SWA Gradient Boost - SWA Manufacturer | -0.91 | 0.04 | 770.33 | -21.84 | 4.79E-81 | all |
| SWA Gradient Boost - SWA Neural Network | -0.04 | 0.04 | 770.01 | -0.93 | 1 | all |
| SWA Gradient Boost - SWA Random Forest | -0.01 | 0.04 | 770.01 | -0.29 | 1 | all |
| SWA Manufacturer - SWA Neural Network | 0.87 | 0.04 | 770.33 | 20.91 | 1.34E-75 | all |
| SWA Manufacturer - SWA Random Forest | 0.90 | 0.04 | 770.33 | 21.55 | 2.48E-79 | all |
| SWA Neural Network - SWA Random Forest | 0.03 | 0.04 | 770.01 | 0.64 | 1 | all |

ADL:

The model was significant (F = 35.69, p < 2.2e-16).

| contrast | estimate | SE | df | t.ratio | p.value | comparison |
| --- | --- | --- | --- | --- | --- | --- |
| AG Gradient Boost - AG Neural Network | -0.15 | 0.08 | 767.12 | -1.74 | 1 | ADL |
| AG Gradient Boost - AG Random Forest | -0.02 | 0.08 | 767.12 | -0.21 | 1 | ADL |
| AG Gradient Boost - FB Gradient Boost | -0.41 | 0.09 | 769.62 | -4.85 | 6.74E-05 | ADL |
| AG Gradient Boost - FB Neural Network | -0.49 | 0.09 | 769.62 | -5.81 | 4.18E-07 | ADL |
| AG Gradient Boost - FB Random Forest | -0.43 | 0.09 | 769.62 | -5.02 | 2.87E-05 | ADL |
| AG Gradient Boost - SWA Gradient Boost | -0.04 | 0.08 | 768.25 | -0.47 | 1 | ADL |
| AG Gradient Boost - SWA Manufacturer | -1.14 | 0.08 | 768.94 | -13.53 | 6.26E-36 | ADL |
| AG Gradient Boost - SWA Neural Network | -0.06 | 0.08 | 768.25 | -0.72 | 1 | ADL |
| AG Gradient Boost - SWA Random Forest | -0.07 | 0.08 | 768.25 | -0.88 | 1 | ADL |
| AG Neural Network - AG Random Forest | 0.13 | 0.08 | 767.12 | 1.52 | 1 | ADL |
| AG Neural Network - FB Gradient Boost | -0.26 | 0.09 | 769.62 | -3.12 | 0.085417 | ADL |
| AG Neural Network - FB Neural Network | -0.35 | 0.09 | 769.62 | -4.07 | 0.002292 | ADL |
| AG Neural Network - FB Random Forest | -0.28 | 0.09 | 769.62 | -3.29 | 0.047461 | ADL |
| AG Neural Network - SWA Gradient Boost | 0.11 | 0.08 | 768.25 | 1.27 | 1 | ADL |
| AG Neural Network - SWA Manufacturer | -1.00 | 0.08 | 768.94 | -11.79 | 5.97E-28 | ADL |
| AG Neural Network - SWA Neural Network | 0.09 | 0.08 | 768.25 | 1.02 | 1 | ADL |
| AG Neural Network - SWA Random Forest | 0.07 | 0.08 | 768.25 | 0.86 | 1 | ADL |
| AG Random Forest - FB Gradient Boost | -0.39 | 0.09 | 769.62 | -4.64 | 0.000188 | ADL |
| AG Random Forest - FB Neural Network | -0.48 | 0.09 | 769.62 | -5.59 | 1.39E-06 | ADL |
| AG Random Forest - FB Random Forest | -0.41 | 0.09 | 769.62 | -4.81 | 8.30E-05 | ADL |
| AG Random Forest - SWA Gradient Boost | -0.02 | 0.08 | 768.25 | -0.26 | 1 | ADL |
| AG Random Forest - SWA Manufacturer | -1.12 | 0.08 | 768.94 | -13.32 | 6.62E-35 | ADL |
| AG Random Forest - SWA Neural Network | -0.04 | 0.08 | 768.25 | -0.50 | 1 | ADL |
| AG Random Forest - SWA Random Forest | -0.06 | 0.08 | 768.25 | -0.66 | 1 | ADL |
| FB Gradient Boost - FB Neural Network | -0.08 | 0.08 | 767.12 | -0.96 | 1 | ADL |
| FB Gradient Boost - FB Random Forest | -0.01 | 0.08 | 767.12 | -0.17 | 1 | ADL |
| FB Gradient Boost - SWA Gradient Boost | 0.37 | 0.08 | 769.33 | 4.39 | 0.000575 | ADL |
| FB Gradient Boost - SWA Manufacturer | -0.73 | 0.08 | 768.59 | -8.65 | 1.31E-15 | ADL |
| FB Gradient Boost - SWA Neural Network | 0.35 | 0.08 | 769.33 | 4.15 | 0.001689 | ADL |
| FB Gradient Boost - SWA Random Forest | 0.34 | 0.08 | 769.33 | 3.99 | 0.00329 | ADL |
| FB Neural Network - FB Random Forest | 0.07 | 0.08 | 767.12 | 0.79 | 1 | ADL |
| FB Neural Network - SWA Gradient Boost | 0.45 | 0.08 | 769.33 | 5.35 | 5.14E-06 | ADL |
| FB Neural Network - SWA Manufacturer | -0.65 | 0.08 | 768.59 | -7.69 | 2.03E-12 | ADL |
| FB Neural Network - SWA Neural Network | 0.43 | 0.08 | 769.33 | 5.11 | 1.86E-05 | ADL |
| FB Neural Network - SWA Random Forest | 0.42 | 0.08 | 769.33 | 4.95 | 4.14E-05 | ADL |
| FB Random Forest - SWA Gradient Boost | 0.39 | 0.08 | 769.33 | 4.56 | 0.000262 | ADL |
| FB Random Forest - SWA Manufacturer | -0.72 | 0.08 | 768.59 | -8.48 | 5.13E-15 | ADL |
| FB Random Forest - SWA Neural Network | 0.37 | 0.08 | 769.33 | 4.32 | 0.000799 | ADL |
| FB Random Forest - SWA Random Forest | 0.35 | 0.08 | 769.33 | 4.16 | 0.001596 | ADL |
| SWA Gradient Boost - SWA Manufacturer | -1.10 | 0.08 | 767.82 | -13.11 | 6.39E-34 | ADL |
| SWA Gradient Boost - SWA Neural Network | -0.02 | 0.08 | 767.12 | -0.25 | 1 | ADL |
| SWA Gradient Boost - SWA Random Forest | -0.03 | 0.08 | 767.12 | -0.41 | 1 | ADL |
| SWA Manufacturer - SWA Neural Network | 1.08 | 0.08 | 767.82 | 12.86 | 9.06E-33 | ADL |
| SWA Manufacturer - SWA Random Forest | 1.07 | 0.08 | 767.82 | 12.70 | 4.94E-32 | ADL |
| SWA Neural Network - SWA Random Forest | -0.01 | 0.08 | 767.12 | -0.16 | 1 | ADL |

Cycling:

The model was significant (F = 51.88, p < 2.2e-16).

| contrast | estimate | SE | df | t.ratio | p.value | comparison |
| --- | --- | --- | --- | --- | --- | --- |
| AG Gradient Boost - AG Neural Network | -0.24 | 0.07 | 770.01 | -3.20 | 0.064555 | Cycling |
| AG Gradient Boost - AG Random Forest | 0.00 | 0.07 | 770.01 | -0.07 | 1 | Cycling |
| AG Gradient Boost - FB Gradient Boost | -0.52 | 0.07 | 770.82 | -7.08 | 1.51E-10 | Cycling |
| AG Gradient Boost - FB Neural Network | -0.66 | 0.07 | 770.82 | -8.95 | 1.21E-16 | Cycling |
| AG Gradient Boost - FB Random Forest | -0.50 | 0.07 | 770.82 | -6.69 | 1.89E-09 | Cycling |
| AG Gradient Boost - SWA Gradient Boost | -0.01 | 0.07 | 770.29 | -0.20 | 1 | Cycling |
| AG Gradient Boost - SWA Manufacturer | -1.10 | 0.07 | 770.60 | -14.99 | 4.15E-43 | Cycling |
| AG Gradient Boost - SWA Neural Network | -0.08 | 0.07 | 770.29 | -1.06 | 1 | Cycling |
| AG Gradient Boost - SWA Random Forest | 0.01 | 0.07 | 770.29 | 0.14 | 1 | Cycling |
| AG Neural Network - AG Random Forest | 0.23 | 0.07 | 770.01 | 3.13 | 0.080914 | Cycling |
| AG Neural Network - FB Gradient Boost | -0.29 | 0.07 | 770.82 | -3.89 | 0.004859 | Cycling |
| AG Neural Network - FB Neural Network | -0.43 | 0.07 | 770.82 | -5.76 | 5.37E-07 | Cycling |
| AG Neural Network - FB Random Forest | -0.26 | 0.07 | 770.82 | -3.51 | 0.021292 | Cycling |
| AG Neural Network - SWA Gradient Boost | 0.22 | 0.07 | 770.29 | 3.00 | 0.12596 | Cycling |
| AG Neural Network - SWA Manufacturer | -0.87 | 0.07 | 770.60 | -11.79 | 6.08E-28 | Cycling |
| AG Neural Network - SWA Neural Network | 0.16 | 0.07 | 770.29 | 2.14 | 1 | Cycling |
| AG Neural Network - SWA Random Forest | 0.25 | 0.07 | 770.29 | 3.33 | 0.040427 | Cycling |
| AG Random Forest - FB Gradient Boost | -0.52 | 0.07 | 770.82 | -7.01 | 2.36E-10 | Cycling |
| AG Random Forest - FB Neural Network | -0.66 | 0.07 | 770.82 | -8.88 | 2.09E-16 | Cycling |
| AG Random Forest - FB Random Forest | -0.49 | 0.07 | 770.82 | -6.63 | 2.89E-09 | Cycling |
| AG Random Forest - SWA Gradient Boost | -0.01 | 0.07 | 770.29 | -0.13 | 1 | Cycling |
| AG Random Forest - SWA Manufacturer | -1.10 | 0.07 | 770.60 | -14.92 | 9.01E-43 | Cycling |
| AG Random Forest - SWA Neural Network | -0.07 | 0.07 | 770.29 | -0.99 | 1 | Cycling |
| AG Random Forest - SWA Random Forest | 0.02 | 0.07 | 770.29 | 0.20 | 1 | Cycling |
| FB Gradient Boost - FB Neural Network | -0.14 | 0.07 | 770.01 | -1.87 | 1 | Cycling |
| FB Gradient Boost - FB Random Forest | 0.03 | 0.07 | 770.01 | 0.38 | 1 | Cycling |
| FB Gradient Boost - SWA Gradient Boost | 0.51 | 0.07 | 770.86 | 6.88 | 5.66E-10 | Cycling |
| FB Gradient Boost - SWA Manufacturer | -0.58 | 0.07 | 770.53 | -7.84 | 6.53E-13 | Cycling |
| FB Gradient Boost - SWA Neural Network | 0.45 | 0.07 | 770.86 | 6.02 | 1.19E-07 | Cycling |
| FB Gradient Boost - SWA Random Forest | 0.53 | 0.07 | 770.86 | 7.21 | 5.97E-11 | Cycling |
| FB Neural Network - FB Random Forest | 0.17 | 0.07 | 770.01 | 2.25 | 1 | Cycling |
| FB Neural Network - SWA Gradient Boost | 0.65 | 0.07 | 770.86 | 8.75 | 6.05E-16 | Cycling |
| FB Neural Network - SWA Manufacturer | -0.44 | 0.07 | 770.53 | -5.97 | 1.66E-07 | Cycling |
| FB Neural Network - SWA Neural Network | 0.59 | 0.07 | 770.86 | 7.89 | 4.53E-13 | Cycling |
| FB Neural Network - SWA Random Forest | 0.67 | 0.07 | 770.86 | 9.08 | 3.96E-17 | Cycling |
| FB Random Forest - SWA Gradient Boost | 0.48 | 0.07 | 770.86 | 6.50 | 6.65E-09 | Cycling |
| FB Random Forest - SWA Manufacturer | -0.61 | 0.07 | 770.53 | -8.23 | 3.66E-14 | Cycling |
| FB Random Forest - SWA Neural Network | 0.42 | 0.07 | 770.86 | 5.64 | 1.07E-06 | Cycling |
| FB Random Forest - SWA Random Forest | 0.51 | 0.07 | 770.86 | 6.83 | 7.77E-10 | Cycling |
| SWA Gradient Boost - SWA Manufacturer | -1.09 | 0.07 | 770.32 | -14.80 | 3.64E-42 | Cycling |
| SWA Gradient Boost - SWA Neural Network | -0.06 | 0.07 | 770.01 | -0.86 | 1 | Cycling |
| SWA Gradient Boost - SWA Random Forest | 0.02 | 0.07 | 770.01 | 0.34 | 1 | Cycling |
| SWA Manufacturer - SWA Neural Network | 1.03 | 0.07 | 770.32 | 13.94 | 6.60E-38 | Cycling |
| SWA Manufacturer - SWA Random Forest | 1.11 | 0.07 | 770.32 | 15.14 | 7.27E-44 | Cycling |
| SWA Neural Network - SWA Random Forest | 0.09 | 0.07 | 770.01 | 1.19 | 1 | Cycling |

Running:

The model was significant (F = 32.469, p < 2.2e-16).

| contrast | estimate | SE | df | t.ratio | p.value | comparison |
| --- | --- | --- | --- | --- | --- | --- |
| AG Gradient Boost - AG Neural Network | -0.32 | 0.08 | 692.00 | -4.20 | 0.001372 | Running |
| AG Gradient Boost - AG Random Forest | -0.01 | 0.08 | 692.00 | -0.08 | 1 | Running |
| AG Gradient Boost - FB Gradient Boost | -0.11 | 0.08 | 692.25 | -1.40 | 1 | Running |
| AG Gradient Boost - FB Neural Network | -0.27 | 0.08 | 692.25 | -3.49 | 0.02346 | Running |
| AG Gradient Boost - FB Random Forest | -0.09 | 0.08 | 692.25 | -1.12 | 1 | Running |
| AG Gradient Boost - SWA Gradient Boost | 0.07 | 0.08 | 692.16 | 0.90 | 1 | Running |
| AG Gradient Boost - SWA Manufacturer | -0.95 | 0.08 | 692.16 | -12.28 | 7.72E-30 | Running |
| AG Gradient Boost - SWA Neural Network | 0.08 | 0.08 | 692.16 | 0.97 | 1 | Running |
| AG Gradient Boost - SWA Random Forest | 0.07 | 0.08 | 692.16 | 0.85 | 1 | Running |
| AG Neural Network - AG Random Forest | 0.32 | 0.08 | 692.00 | 4.11 | 0.00197 | Running |
| AG Neural Network - FB Gradient Boost | 0.22 | 0.08 | 692.25 | 2.77 | 0.259469 | Running |
| AG Neural Network - FB Neural Network | 0.05 | 0.08 | 692.25 | 0.68 | 1 | Running |
| AG Neural Network - FB Random Forest | 0.24 | 0.08 | 692.25 | 3.05 | 0.107199 | Running |
| AG Neural Network - SWA Gradient Boost | 0.39 | 0.08 | 692.16 | 5.08 | 2.17E-05 | Running |
| AG Neural Network - SWA Manufacturer | -0.63 | 0.08 | 692.16 | -8.10 | 1.15E-13 | Running |
| AG Neural Network - SWA Neural Network | 0.40 | 0.08 | 692.16 | 5.15 | 1.50E-05 | Running |
| AG Neural Network - SWA Random Forest | 0.39 | 0.08 | 692.16 | 5.03 | 2.84E-05 | Running |
| AG Random Forest - FB Gradient Boost | -0.10 | 0.08 | 692.25 | -1.31 | 1 | Running |
| AG Random Forest - FB Neural Network | -0.26 | 0.08 | 692.25 | -3.40 | 0.031851 | Running |
| AG Random Forest - FB Random Forest | -0.08 | 0.08 | 692.25 | -1.03 | 1 | Running |
| AG Random Forest - SWA Gradient Boost | 0.08 | 0.08 | 692.16 | 0.98 | 1 | Running |
| AG Random Forest - SWA Manufacturer | -0.94 | 0.08 | 692.16 | -12.19 | 1.82E-29 | Running |
| AG Random Forest - SWA Neural Network | 0.08 | 0.08 | 692.16 | 1.06 | 1 | Running |
| AG Random Forest - SWA Random Forest | 0.07 | 0.08 | 692.16 | 0.93 | 1 | Running |
| FB Gradient Boost - FB Neural Network | -0.16 | 0.08 | 692.00 | -2.08 | 1 | Running |
| FB Gradient Boost - FB Random Forest | 0.02 | 0.08 | 692.00 | 0.28 | 1 | Running |
| FB Gradient Boost - SWA Gradient Boost | 0.18 | 0.08 | 692.42 | 2.29 | 1 | Running |
| FB Gradient Boost - SWA Manufacturer | -0.84 | 0.08 | 692.42 | -10.80 | 1.43E-23 | Running |
| FB Gradient Boost - SWA Neural Network | 0.18 | 0.08 | 692.42 | 2.36 | 0.839723 | Running |
| FB Gradient Boost - SWA Random Forest | 0.17 | 0.08 | 692.42 | 2.23 | 1 | Running |
| FB Neural Network - FB Random Forest | 0.18 | 0.08 | 692.00 | 2.35 | 0.84621 | Running |
| FB Neural Network - SWA Gradient Boost | 0.34 | 0.08 | 692.42 | 4.37 | 0.000657 | Running |
| FB Neural Network - SWA Manufacturer | -0.68 | 0.08 | 692.42 | -8.72 | 9.49E-16 | Running |
| FB Neural Network - SWA Neural Network | 0.35 | 0.08 | 692.42 | 4.44 | 0.000474 | Running |
| FB Neural Network - SWA Random Forest | 0.34 | 0.08 | 692.42 | 4.31 | 0.00083 | Running |
| FB Random Forest - SWA Gradient Boost | 0.16 | 0.08 | 692.42 | 2.01 | 1 | Running |
| FB Random Forest - SWA Manufacturer | -0.86 | 0.08 | 692.42 | -11.08 | 1.04E-24 | Running |
| FB Random Forest - SWA Neural Network | 0.16 | 0.08 | 692.42 | 2.08 | 1 | Running |
| FB Random Forest - SWA Random Forest | 0.15 | 0.08 | 692.42 | 1.95 | 1 | Running |
| SWA Gradient Boost - SWA Manufacturer | -1.02 | 0.08 | 692.00 | -13.14 | 1.00E-33 | Running |
| SWA Gradient Boost - SWA Neural Network | 0.01 | 0.08 | 692.00 | 0.07 | 1 | Running |
| SWA Gradient Boost - SWA Random Forest | 0.00 | 0.08 | 692.00 | -0.05 | 1 | Running |
| SWA Manufacturer - SWA Neural Network | 1.03 | 0.08 | 692.00 | 13.22 | 4.63E-34 | Running |
| SWA Manufacturer - SWA Random Forest | 1.02 | 0.08 | 692.00 | 13.09 | 1.75E-33 | Running |
| SWA Neural Network - SWA Random Forest | -0.01 | 0.08 | 692.00 | -0.13 | 1 | Running |

Sedentary:

The model was significant (F = 9.8229, p = 2.517e-14).

| contrast | estimate | SE | df | t.ratio | p.value | comparison |
| --- | --- | --- | --- | --- | --- | --- |
| AG Gradient Boost - AG Neural Network | -0.09 | 0.03 | 767.04 | -2.56 | 0.475947 | Sedentary |
| AG Gradient Boost - AG Random Forest | 0.00 | 0.03 | 767.04 | -0.15 | 1 | Sedentary |
| AG Gradient Boost - FB Gradient Boost | -0.18 | 0.03 | 768.60 | -5.38 | 4.43E-06 | Sedentary |
| AG Gradient Boost - FB Neural Network | -0.20 | 0.03 | 768.60 | -6.07 | 9.06E-08 | Sedentary |
| AG Gradient Boost - FB Random Forest | -0.16 | 0.03 | 768.60 | -4.76 | 0.000103 | Sedentary |
| AG Gradient Boost - SWA Gradient Boost | -0.02 | 0.03 | 767.72 | -0.54 | 1 | Sedentary |
| AG Gradient Boost - SWA Manufacturer | -0.09 | 0.03 | 768.20 | -2.66 | 0.355831 | Sedentary |
| AG Gradient Boost - SWA Neural Network | -0.08 | 0.03 | 767.72 | -2.36 | 0.832649 | Sedentary |
| AG Gradient Boost - SWA Random Forest | -0.03 | 0.03 | 767.72 | -0.88 | 1 | Sedentary |
| AG Neural Network - AG Random Forest | 0.08 | 0.03 | 767.04 | 2.42 | 0.717365 | Sedentary |
| AG Neural Network - FB Gradient Boost | -0.09 | 0.03 | 768.60 | -2.82 | 0.218923 | Sedentary |
| AG Neural Network - FB Neural Network | -0.12 | 0.03 | 768.60 | -3.51 | 0.021111 | Sedentary |
| AG Neural Network - FB Random Forest | -0.07 | 0.03 | 768.60 | -2.21 | 1 | Sedentary |
| AG Neural Network - SWA Gradient Boost | 0.07 | 0.03 | 767.72 | 2.03 | 1 | Sedentary |
| AG Neural Network - SWA Manufacturer | 0.00 | 0.03 | 768.20 | -0.09 | 1 | Sedentary |
| AG Neural Network - SWA Neural Network | 0.01 | 0.03 | 767.72 | 0.21 | 1 | Sedentary |
| AG Neural Network - SWA Random Forest | 0.06 | 0.03 | 767.72 | 1.69 | 1 | Sedentary |
| AG Random Forest - FB Gradient Boost | -0.17 | 0.03 | 768.60 | -5.23 | 9.64E-06 | Sedentary |
| AG Random Forest - FB Neural Network | -0.20 | 0.03 | 768.60 | -5.92 | 2.15E-07 | Sedentary |
| AG Random Forest - FB Random Forest | -0.15 | 0.03 | 768.60 | -4.62 | 0.000206 | Sedentary |
| AG Random Forest - SWA Gradient Boost | -0.01 | 0.03 | 767.72 | -0.39 | 1 | Sedentary |
| AG Random Forest - SWA Manufacturer | -0.08 | 0.03 | 768.20 | -2.52 | 0.544308 | Sedentary |
| AG Random Forest - SWA Neural Network | -0.07 | 0.03 | 767.72 | -2.21 | 1 | Sedentary |
| AG Random Forest - SWA Random Forest | -0.02 | 0.03 | 767.72 | -0.73 | 1 | Sedentary |
| FB Gradient Boost - FB Neural Network | -0.02 | 0.03 | 767.04 | -0.69 | 1 | Sedentary |
| FB Gradient Boost - FB Random Forest | 0.02 | 0.03 | 767.04 | 0.62 | 1 | Sedentary |
| FB Gradient Boost - SWA Gradient Boost | 0.16 | 0.03 | 768.44 | 4.86 | 6.51E-05 | Sedentary |
| FB Gradient Boost - SWA Manufacturer | 0.09 | 0.03 | 767.92 | 2.75 | 0.272076 | Sedentary |
| FB Gradient Boost - SWA Neural Network | 0.10 | 0.03 | 768.44 | 3.04 | 0.110165 | Sedentary |
| FB Gradient Boost - SWA Random Forest | 0.15 | 0.03 | 768.44 | 4.52 | 0.000318 | Sedentary |
| FB Neural Network - FB Random Forest | 0.04 | 0.03 | 767.04 | 1.31 | 1 | Sedentary |
| FB Neural Network - SWA Gradient Boost | 0.18 | 0.03 | 768.44 | 5.55 | 1.79E-06 | Sedentary |
| FB Neural Network - SWA Manufacturer | 0.11 | 0.03 | 767.92 | 3.45 | 0.026962 | Sedentary |
| FB Neural Network - SWA Neural Network | 0.12 | 0.03 | 768.44 | 3.73 | 0.009221 | Sedentary |
| FB Neural Network - SWA Random Forest | 0.17 | 0.03 | 768.44 | 5.21 | 1.07E-05 | Sedentary |
| FB Random Forest - SWA Gradient Boost | 0.14 | 0.03 | 768.44 | 4.24 | 0.001145 | Sedentary |
| FB Random Forest - SWA Manufacturer | 0.07 | 0.03 | 767.92 | 2.13 | 1 | Sedentary |
| FB Random Forest - SWA Neural Network | 0.08 | 0.03 | 768.44 | 2.42 | 0.709673 | Sedentary |
| FB Random Forest - SWA Random Forest | 0.13 | 0.03 | 768.44 | 3.90 | 0.004652 | Sedentary |
| SWA Gradient Boost - SWA Manufacturer | -0.07 | 0.03 | 767.53 | -2.13 | 1 | Sedentary |
| SWA Gradient Boost - SWA Neural Network | -0.06 | 0.03 | 767.04 | -1.83 | 1 | Sedentary |
| SWA Gradient Boost - SWA Random Forest | -0.01 | 0.03 | 767.04 | -0.34 | 1 | Sedentary |
| SWA Manufacturer - SWA Neural Network | 0.01 | 0.03 | 767.53 | 0.30 | 1 | Sedentary |
| SWA Manufacturer - SWA Random Forest | 0.06 | 0.03 | 767.53 | 1.79 | 1 | Sedentary |
| SWA Neural Network - SWA Random Forest | 0.05 | 0.03 | 767.04 | 1.49 | 1 | Sedentary |

Transitional:

The model was significant (F = 22.83, p = p < 2.2e-16).

| contrast | estimate | SE | df | t.ratio | p.value | comparison |
| --- | --- | --- | --- | --- | --- | --- |
| AG Gradient Boost - AG Neural Network | -0.31 | 0.07 | 701.02 | -4.19 | 0.001411 | Transitional |
| AG Gradient Boost - AG Random Forest | -0.03 | 0.07 | 701.02 | -0.40 | 1 | Transitional |
| AG Gradient Boost - FB Gradient Boost | -0.38 | 0.07 | 701.76 | -5.15 | 1.53E-05 | Transitional |
| AG Gradient Boost - FB Neural Network | -0.51 | 0.07 | 701.76 | -6.87 | 6.52E-10 | Transitional |
| AG Gradient Boost - FB Random Forest | -0.40 | 0.07 | 701.76 | -5.32 | 6.22E-06 | Transitional |
| AG Gradient Boost - SWA Gradient Boost | -0.02 | 0.07 | 701.32 | -0.23 | 1 | Transitional |
| AG Gradient Boost - SWA Manufacturer | -0.71 | 0.07 | 701.66 | -9.63 | 4.59E-19 | Transitional |
| AG Gradient Boost - SWA Neural Network | -0.10 | 0.07 | 701.32 | -1.39 | 1 | Transitional |
| AG Gradient Boost - SWA Random Forest | -0.02 | 0.07 | 701.32 | -0.22 | 1 | Transitional |
| AG Neural Network - AG Random Forest | 0.28 | 0.07 | 701.02 | 3.79 | 0.007456 | Transitional |
| AG Neural Network - FB Gradient Boost | -0.07 | 0.07 | 701.76 | -0.97 | 1 | Transitional |
| AG Neural Network - FB Neural Network | -0.20 | 0.07 | 701.76 | -2.68 | 0.335217 | Transitional |
| AG Neural Network - FB Random Forest | -0.08 | 0.07 | 701.76 | -1.14 | 1 | Transitional |
| AG Neural Network - SWA Gradient Boost | 0.29 | 0.07 | 701.32 | 3.96 | 0.003742 | Transitional |
| AG Neural Network - SWA Manufacturer | -0.40 | 0.07 | 701.66 | -5.44 | 3.36E-06 | Transitional |
| AG Neural Network - SWA Neural Network | 0.21 | 0.07 | 701.32 | 2.80 | 0.237484 | Transitional |
| AG Neural Network - SWA Random Forest | 0.29 | 0.07 | 701.32 | 3.97 | 0.003567 | Transitional |
| AG Random Forest - FB Gradient Boost | -0.35 | 0.07 | 701.76 | -4.75 | 0.000113 | Transitional |
| AG Random Forest - FB Neural Network | -0.48 | 0.07 | 701.76 | -6.46 | 8.62E-09 | Transitional |
| AG Random Forest - FB Random Forest | -0.37 | 0.07 | 701.76 | -4.92 | 4.89E-05 | Transitional |
| AG Random Forest - SWA Gradient Boost | 0.01 | 0.07 | 701.32 | 0.17 | 1 | Transitional |
| AG Random Forest - SWA Manufacturer | -0.68 | 0.07 | 701.66 | -9.23 | 1.41E-17 | Transitional |
| AG Random Forest - SWA Neural Network | -0.07 | 0.07 | 701.32 | -0.99 | 1 | Transitional |
| AG Random Forest - SWA Random Forest | 0.01 | 0.07 | 701.32 | 0.19 | 1 | Transitional |
| FB Gradient Boost - FB Neural Network | -0.13 | 0.07 | 701.02 | -1.72 | 1 | Transitional |
| FB Gradient Boost - FB Random Forest | -0.01 | 0.07 | 701.02 | -0.17 | 1 | Transitional |
| FB Gradient Boost - SWA Gradient Boost | 0.37 | 0.07 | 701.80 | 4.92 | 4.84E-05 | Transitional |
| FB Gradient Boost - SWA Manufacturer | -0.33 | 0.07 | 701.45 | -4.47 | 0.000413 | Transitional |
| FB Gradient Boost - SWA Neural Network | 0.28 | 0.07 | 701.80 | 3.76 | 0.008228 | Transitional |
| FB Gradient Boost - SWA Random Forest | 0.37 | 0.07 | 701.80 | 4.93 | 4.57E-05 | Transitional |
| FB Neural Network - FB Random Forest | 0.11 | 0.07 | 701.02 | 1.55 | 1 | Transitional |
| FB Neural Network - SWA Gradient Boost | 0.49 | 0.07 | 701.80 | 6.64 | 2.87E-09 | Transitional |
| FB Neural Network - SWA Manufacturer | -0.20 | 0.07 | 701.45 | -2.74 | 0.279499 | Transitional |
| FB Neural Network - SWA Neural Network | 0.41 | 0.07 | 701.80 | 5.48 | 2.68E-06 | Transitional |
| FB Neural Network - SWA Random Forest | 0.49 | 0.07 | 701.80 | 6.65 | 2.66E-09 | Transitional |
| FB Random Forest - SWA Gradient Boost | 0.38 | 0.07 | 701.80 | 5.09 | 2.04E-05 | Transitional |
| FB Random Forest - SWA Manufacturer | -0.32 | 0.07 | 701.45 | -4.30 | 0.000896 | Transitional |
| FB Random Forest - SWA Neural Network | 0.29 | 0.07 | 701.80 | 3.93 | 0.004127 | Transitional |
| FB Random Forest - SWA Random Forest | 0.38 | 0.07 | 701.80 | 5.10 | 1.92E-05 | Transitional |
| SWA Gradient Boost - SWA Manufacturer | -0.70 | 0.07 | 701.35 | -9.41 | 3.03E-18 | Transitional |
| SWA Gradient Boost - SWA Neural Network | -0.09 | 0.07 | 701.02 | -1.16 | 1 | Transitional |
| SWA Gradient Boost - SWA Random Forest | 0.00 | 0.07 | 701.02 | 0.01 | 1 | Transitional |
| SWA Manufacturer - SWA Neural Network | 0.61 | 0.07 | 701.35 | 8.25 | 3.53E-14 | Transitional |
| SWA Manufacturer - SWA Random Forest | 0.70 | 0.07 | 701.35 | 9.43 | 2.74E-18 | Transitional |
| SWA Neural Network - SWA Random Forest | 0.09 | 0.07 | 701.02 | 1.17 | 1 | Transitional |

Walking:

The model was significant (F = 18.49, p = p < 2.2e-16).

| contrast | estimate | SE | df | t.ratio | p.value | comparison |
| --- | --- | --- | --- | --- | --- | --- |
| AG Gradient Boost - AG Neural Network | -0.16 | 0.05 | 770.04 | -2.96 | 0.144591 | Walking |
| AG Gradient Boost - AG Random Forest | -0.01 | 0.05 | 770.04 | -0.15 | 1 | Walking |
| AG Gradient Boost - FB Gradient Boost | -0.18 | 0.05 | 771.42 | -3.32 | 0.043128 | Walking |
| AG Gradient Boost - FB Neural Network | -0.29 | 0.05 | 771.42 | -5.38 | 4.47E-06 | Walking |
| AG Gradient Boost - FB Random Forest | -0.18 | 0.05 | 771.42 | -3.39 | 0.032612 | Walking |
| AG Gradient Boost - SWA Gradient Boost | 0.06 | 0.05 | 770.53 | 1.20 | 1 | Walking |
| AG Gradient Boost - SWA Manufacturer | -0.42 | 0.05 | 771.02 | -7.99 | 2.21E-13 | Walking |
| AG Gradient Boost - SWA Neural Network | -0.01 | 0.05 | 770.53 | -0.19 | 1 | Walking |
| AG Gradient Boost - SWA Random Forest | 0.07 | 0.05 | 770.53 | 1.25 | 1 | Walking |
| AG Neural Network - AG Random Forest | 0.15 | 0.05 | 770.04 | 2.81 | 0.229032 | Walking |
| AG Neural Network - FB Gradient Boost | -0.02 | 0.05 | 771.42 | -0.37 | 1 | Walking |
| AG Neural Network - FB Neural Network | -0.13 | 0.05 | 771.42 | -2.44 | 0.675666 | Walking |
| AG Neural Network - FB Random Forest | -0.02 | 0.05 | 771.42 | -0.45 | 1 | Walking |
| AG Neural Network - SWA Gradient Boost | 0.22 | 0.05 | 770.53 | 4.15 | 0.001664 | Walking |
| AG Neural Network - SWA Manufacturer | -0.27 | 0.05 | 771.02 | -5.03 | 2.76E-05 | Walking |
| AG Neural Network - SWA Neural Network | 0.15 | 0.05 | 770.53 | 2.76 | 0.263524 | Walking |
| AG Neural Network - SWA Random Forest | 0.22 | 0.05 | 770.53 | 4.21 | 0.001294 | Walking |
| AG Random Forest - FB Gradient Boost | -0.17 | 0.05 | 771.42 | -3.17 | 0.071437 | Walking |
| AG Random Forest - FB Neural Network | -0.28 | 0.05 | 771.42 | -5.23 | 9.67E-06 | Walking |
| AG Random Forest - FB Random Forest | -0.17 | 0.05 | 771.42 | -3.25 | 0.054574 | Walking |
| AG Random Forest - SWA Gradient Boost | 0.07 | 0.05 | 770.53 | 1.34 | 1 | Walking |
| AG Random Forest - SWA Manufacturer | -0.41 | 0.05 | 771.02 | -7.84 | 6.59E-13 | Walking |
| AG Random Forest - SWA Neural Network | 0.00 | 0.05 | 770.53 | -0.04 | 1 | Walking |
| AG Random Forest - SWA Random Forest | 0.07 | 0.05 | 770.53 | 1.40 | 1 | Walking |
| FB Gradient Boost - FB Neural Network | -0.11 | 0.05 | 770.04 | -2.06 | 1 | Walking |
| FB Gradient Boost - FB Random Forest | 0.00 | 0.05 | 770.04 | -0.08 | 1 | Walking |
| FB Gradient Boost - SWA Gradient Boost | 0.24 | 0.05 | 771.49 | 4.51 | 0.000343 | Walking |
| FB Gradient Boost - SWA Manufacturer | -0.25 | 0.05 | 770.95 | -4.64 | 0.000185 | Walking |
| FB Gradient Boost - SWA Neural Network | 0.17 | 0.05 | 771.49 | 3.13 | 0.082837 | Walking |
| FB Gradient Boost - SWA Random Forest | 0.24 | 0.05 | 771.49 | 4.56 | 0.000262 | Walking |
| FB Neural Network - FB Random Forest | 0.11 | 0.05 | 770.04 | 1.98 | 1 | Walking |
| FB Neural Network - SWA Gradient Boost | 0.35 | 0.05 | 771.49 | 6.57 | 4.16E-09 | Walking |
| FB Neural Network - SWA Manufacturer | -0.14 | 0.05 | 770.95 | -2.57 | 0.468569 | Walking |
| FB Neural Network - SWA Neural Network | 0.28 | 0.05 | 771.49 | 5.19 | 1.22E-05 | Walking |
| FB Neural Network - SWA Random Forest | 0.35 | 0.05 | 771.49 | 6.63 | 2.87E-09 | Walking |
| FB Random Forest - SWA Gradient Boost | 0.24 | 0.05 | 771.49 | 4.58 | 0.000239 | Walking |
| FB Random Forest - SWA Manufacturer | -0.24 | 0.05 | 770.95 | -4.56 | 0.000267 | Walking |
| FB Random Forest - SWA Neural Network | 0.17 | 0.05 | 771.49 | 3.20 | 0.063479 | Walking |
| FB Random Forest - SWA Random Forest | 0.25 | 0.05 | 771.49 | 4.64 | 0.000182 | Walking |
| SWA Gradient Boost - SWA Manufacturer | -0.49 | 0.05 | 770.54 | -9.20 | 1.54E-17 | Walking |
| SWA Gradient Boost - SWA Neural Network | -0.07 | 0.05 | 770.04 | -1.39 | 1 | Walking |
| SWA Gradient Boost - SWA Random Forest | 0.00 | 0.05 | 770.04 | 0.06 | 1 | Walking |
| SWA Manufacturer - SWA Neural Network | 0.41 | 0.05 | 770.54 | 7.80 | 8.74E-13 | Walking |
| SWA Manufacturer - SWA Random Forest | 0.49 | 0.05 | 770.54 | 9.25 | 9.41E-18 | Walking |
| SWA Neural Network - SWA Random Forest | 0.08 | 0.05 | 770.04 | 1.45 | 1 | Walking |

Elliptical:

The model was significant (F = 12.14, p = 7.705e-16).

| contrast | estimate | SE | df | t.ratio | p.value | comparison |
| --- | --- | --- | --- | --- | --- | --- |
| AG Gradient Boost - AG Neural Network | -0.18 | 0.14 | 245.00 | -1.30 | 1 | Elliptical |
| AG Gradient Boost - AG Random Forest | 0.00 | 0.14 | 245.00 | -0.03 | 1 | Elliptical |
| AG Gradient Boost - FB Gradient Boost | -0.47 | 0.14 | 245.07 | -3.38 | 0.03732 | Elliptical |
| AG Gradient Boost - FB Neural Network | -0.61 | 0.14 | 245.07 | -4.39 | 0.000743 | Elliptical |
| AG Gradient Boost - FB Random Forest | -0.41 | 0.14 | 245.07 | -2.93 | 0.166907 | Elliptical |
| AG Gradient Boost - SWA Gradient Boost | 0.12 | 0.14 | 245.09 | 0.84 | 1 | Elliptical |
| AG Gradient Boost - SWA Manufacturer | -0.89 | 0.14 | 245.09 | -6.40 | 3.61E-08 | Elliptical |
| AG Gradient Boost - SWA Neural Network | 0.01 | 0.14 | 245.09 | 0.10 | 1 | Elliptical |
| AG Gradient Boost - SWA Random Forest | 0.11 | 0.14 | 245.09 | 0.80 | 1 | Elliptical |
| AG Neural Network - AG Random Forest | 0.17 | 0.14 | 245.00 | 1.27 | 1 | Elliptical |
| AG Neural Network - FB Gradient Boost | -0.29 | 0.14 | 245.07 | -2.10 | 1 | Elliptical |
| AG Neural Network - FB Neural Network | -0.43 | 0.14 | 245.07 | -3.11 | 0.094231 | Elliptical |
| AG Neural Network - FB Random Forest | -0.23 | 0.14 | 245.07 | -1.64 | 1 | Elliptical |
| AG Neural Network - SWA Gradient Boost | 0.30 | 0.14 | 245.09 | 2.12 | 1 | Elliptical |
| AG Neural Network - SWA Manufacturer | -0.71 | 0.14 | 245.09 | -5.11 | 2.91E-05 | Elliptical |
| AG Neural Network - SWA Neural Network | 0.19 | 0.14 | 245.09 | 1.38 | 1 | Elliptical |
| AG Neural Network - SWA Random Forest | 0.29 | 0.14 | 245.09 | 2.09 | 1 | Elliptical |
| AG Random Forest - FB Gradient Boost | -0.47 | 0.14 | 245.07 | -3.36 | 0.041189 | Elliptical |
| AG Random Forest - FB Neural Network | -0.61 | 0.14 | 245.07 | -4.37 | 0.000839 | Elliptical |
| AG Random Forest - FB Random Forest | -0.40 | 0.14 | 245.07 | -2.90 | 0.182273 | Elliptical |
| AG Random Forest - SWA Gradient Boost | 0.12 | 0.14 | 245.09 | 0.87 | 1 | Elliptical |
| AG Random Forest - SWA Manufacturer | -0.89 | 0.14 | 245.09 | -6.37 | 4.24E-08 | Elliptical |
| AG Random Forest - SWA Neural Network | 0.02 | 0.14 | 245.09 | 0.12 | 1 | Elliptical |
| AG Random Forest - SWA Random Forest | 0.12 | 0.14 | 245.09 | 0.83 | 1 | Elliptical |
| FB Gradient Boost - FB Neural Network | -0.14 | 0.14 | 245.00 | -1.00 | 1 | Elliptical |
| FB Gradient Boost - FB Random Forest | 0.06 | 0.14 | 245.00 | 0.45 | 1 | Elliptical |
| FB Gradient Boost - SWA Gradient Boost | 0.59 | 0.14 | 245.16 | 4.18 | 0.001823 | Elliptical |
| FB Gradient Boost - SWA Manufacturer | -0.42 | 0.14 | 245.16 | -2.98 | 0.142019 | Elliptical |
| FB Gradient Boost - SWA Neural Network | 0.48 | 0.14 | 245.16 | 3.45 | 0.030129 | Elliptical |
| FB Gradient Boost - SWA Random Forest | 0.58 | 0.14 | 245.16 | 4.14 | 0.00212 | Elliptical |
| FB Neural Network - FB Random Forest | 0.20 | 0.14 | 245.00 | 1.45 | 1 | Elliptical |
| FB Neural Network - SWA Gradient Boost | 0.73 | 0.14 | 245.16 | 5.18 | 2.08E-05 | Elliptical |
| FB Neural Network - SWA Manufacturer | -0.28 | 0.14 | 245.16 | -1.98 | 1 | Elliptical |
| FB Neural Network - SWA Neural Network | 0.62 | 0.14 | 245.16 | 4.45 | 0.000598 | Elliptical |
| FB Neural Network - SWA Random Forest | 0.72 | 0.14 | 245.16 | 5.14 | 2.49E-05 | Elliptical |
| FB Random Forest - SWA Gradient Boost | 0.52 | 0.14 | 245.16 | 3.73 | 0.010697 | Elliptical |
| FB Random Forest - SWA Manufacturer | -0.48 | 0.14 | 245.16 | -3.43 | 0.031661 | Elliptical |
| FB Random Forest - SWA Neural Network | 0.42 | 0.14 | 245.16 | 3.00 | 0.135846 | Elliptical |
| FB Random Forest - SWA Random Forest | 0.52 | 0.14 | 245.16 | 3.69 | 0.012278 | Elliptical |
| SWA Gradient Boost - SWA Manufacturer | -1.01 | 0.14 | 245.00 | -7.18 | 3.71E-10 | Elliptical |
| SWA Gradient Boost - SWA Neural Network | -0.10 | 0.14 | 245.00 | -0.74 | 1 | Elliptical |
| SWA Gradient Boost - SWA Random Forest | -0.01 | 0.14 | 245.00 | -0.04 | 1 | Elliptical |
| SWA Manufacturer - SWA Neural Network | 0.90 | 0.14 | 245.00 | 6.44 | 2.74E-08 | Elliptical |
| SWA Manufacturer - SWA Random Forest | 1.00 | 0.14 | 245.00 | 7.14 | 4.64E-10 | Elliptical |
| SWA Neural Network - SWA Random Forest | 0.10 | 0.14 | 245.00 | 0.70 | 1 | Elliptical |

Rowing:

The model was significant (F = 20.7, p = p < 2.2e-16).

| contrast | estimate | SE | df | t.ratio | p.value | comparison |
| --- | --- | --- | --- | --- | --- | --- |
| AG Gradient Boost - AG Neural Network | -0.15 | 0.16 | 254.00 | -0.90 | 1 | Rowing |
| AG Gradient Boost - AG Random Forest | 0.00 | 0.16 | 254.00 | -0.02 | 1 | Rowing |
| AG Gradient Boost - FB Gradient Boost | -0.99 | 0.16 | 254.12 | -6.03 | 2.54E-07 | Rowing |
| AG Gradient Boost - FB Neural Network | -0.91 | 0.16 | 254.12 | -5.55 | 3.19E-06 | Rowing |
| AG Gradient Boost - FB Random Forest | -0.99 | 0.16 | 254.12 | -6.04 | 2.47E-07 | Rowing |
| AG Gradient Boost - SWA Gradient Boost | -0.03 | 0.16 | 254.15 | -0.17 | 1 | Rowing |
| AG Gradient Boost - SWA Manufacturer | -1.33 | 0.16 | 254.15 | -8.10 | 1.03E-12 | Rowing |
| AG Gradient Boost - SWA Neural Network | -0.10 | 0.16 | 254.15 | -0.59 | 1 | Rowing |
| AG Gradient Boost - SWA Random Forest | -0.03 | 0.16 | 254.15 | -0.21 | 1 | Rowing |
| AG Neural Network - AG Random Forest | 0.14 | 0.16 | 254.00 | 0.88 | 1 | Rowing |
| AG Neural Network - FB Gradient Boost | -0.84 | 0.16 | 254.12 | -5.14 | 2.49E-05 | Rowing |
| AG Neural Network - FB Neural Network | -0.77 | 0.16 | 254.12 | -4.66 | 0.000234 | Rowing |
| AG Neural Network - FB Random Forest | -0.85 | 0.16 | 254.12 | -5.14 | 2.44E-05 | Rowing |
| AG Neural Network - SWA Gradient Boost | 0.12 | 0.16 | 254.15 | 0.73 | 1 | Rowing |
| AG Neural Network - SWA Manufacturer | -1.19 | 0.16 | 254.15 | -7.21 | 2.96E-10 | Rowing |
| AG Neural Network - SWA Neural Network | 0.05 | 0.16 | 254.15 | 0.30 | 1 | Rowing |
| AG Neural Network - SWA Random Forest | 0.11 | 0.16 | 254.15 | 0.68 | 1 | Rowing |
| AG Random Forest - FB Gradient Boost | -0.99 | 0.16 | 254.12 | -6.01 | 2.84E-07 | Rowing |
| AG Random Forest - FB Neural Network | -0.91 | 0.16 | 254.12 | -5.53 | 3.56E-06 | Rowing |
| AG Random Forest - FB Random Forest | -0.99 | 0.16 | 254.12 | -6.02 | 2.77E-07 | Rowing |
| AG Random Forest - SWA Gradient Boost | -0.02 | 0.16 | 254.15 | -0.15 | 1 | Rowing |
| AG Random Forest - SWA Manufacturer | -1.33 | 0.16 | 254.15 | -8.08 | 1.18E-12 | Rowing |
| AG Random Forest - SWA Neural Network | -0.09 | 0.16 | 254.15 | -0.57 | 1 | Rowing |
| AG Random Forest - SWA Random Forest | -0.03 | 0.16 | 254.15 | -0.19 | 1 | Rowing |
| FB Gradient Boost - FB Neural Network | 0.08 | 0.17 | 254.00 | 0.48 | 1 | Rowing |
| FB Gradient Boost - FB Random Forest | 0.00 | 0.17 | 254.00 | 0.00 | 1 | Rowing |
| FB Gradient Boost - SWA Gradient Boost | 0.96 | 0.17 | 254.28 | 5.81 | 8.50E-07 | Rowing |
| FB Gradient Boost - SWA Manufacturer | -0.34 | 0.17 | 254.28 | -2.05 | 1 | Rowing |
| FB Gradient Boost - SWA Neural Network | 0.89 | 0.17 | 254.28 | 5.39 | 7.33E-06 | Rowing |
| FB Gradient Boost - SWA Random Forest | 0.96 | 0.17 | 254.28 | 5.77 | 1.06E-06 | Rowing |
| FB Neural Network - FB Random Forest | -0.08 | 0.17 | 254.00 | -0.48 | 1 | Rowing |
| FB Neural Network - SWA Gradient Boost | 0.89 | 0.17 | 254.28 | 5.33 | 9.73E-06 | Rowing |
| FB Neural Network - SWA Manufacturer | -0.42 | 0.17 | 254.28 | -2.53 | 0.547133 | Rowing |
| FB Neural Network - SWA Neural Network | 0.82 | 0.17 | 254.28 | 4.91 | 7.32E-05 | Rowing |
| FB Neural Network - SWA Random Forest | 0.88 | 0.17 | 254.28 | 5.29 | 1.20E-05 | Rowing |
| FB Random Forest - SWA Gradient Boost | 0.97 | 0.17 | 254.28 | 5.81 | 8.28E-07 | Rowing |
| FB Random Forest - SWA Manufacturer | -0.34 | 0.17 | 254.28 | -2.04 | 1 | Rowing |
| FB Random Forest - SWA Neural Network | 0.90 | 0.17 | 254.28 | 5.39 | 7.16E-06 | Rowing |
| FB Random Forest - SWA Random Forest | 0.96 | 0.17 | 254.28 | 5.77 | 1.03E-06 | Rowing |
| SWA Gradient Boost - SWA Manufacturer | -1.30 | 0.17 | 254.00 | -7.88 | 4.46E-12 | Rowing |
| SWA Gradient Boost - SWA Neural Network | -0.07 | 0.17 | 254.00 | -0.42 | 1 | Rowing |
| SWA Gradient Boost - SWA Random Forest | -0.01 | 0.17 | 254.00 | -0.04 | 1 | Rowing |
| SWA Manufacturer - SWA Neural Network | 1.23 | 0.17 | 254.00 | 7.45 | 6.40E-11 | Rowing |
| SWA Manufacturer - SWA Random Forest | 1.30 | 0.17 | 254.00 | 7.83 | 5.84E-12 | Rowing |
| SWA Neural Network - SWA Random Forest | 0.06 | 0.17 | 254.00 | 0.38 | 1 | Rowing |
