# Supplementary material for: Comparison of the Validity and Generalizability of Machine Learning Algorithms for the Prediction of Energy Expenditure: Validation Study
Source: JMIR Mhealth Uhealth. 2021 Aug 4;9(8):e23938. doi: 10.2196/23938 (PMC8374660; doi:10.2196/23938)
Supplement: Multimedia Appendix 4 [file mhealth_v9i8e23938_app4.docx]

**Multimedia Appendix 4.** Permutation importance analysis for Fitbit, SenseWear, and Actigraph datasets.

**Random forest permutation importance**

Permutation importance is computed for random forests using the sklearn.inspection.permutation_importance class. A single random forest model is fitted for the Fitbit dataset, the SenseWear dataset and the Actigraph dataset. A baseline metric is determined for the estimator (R^2^ of the prediction). After a feature is permuted the metric is calculated again, the outcome is the difference between these two scores. The boxplots below represent the most important features according to this measure from 20 iterations.

**Fitbit**

**
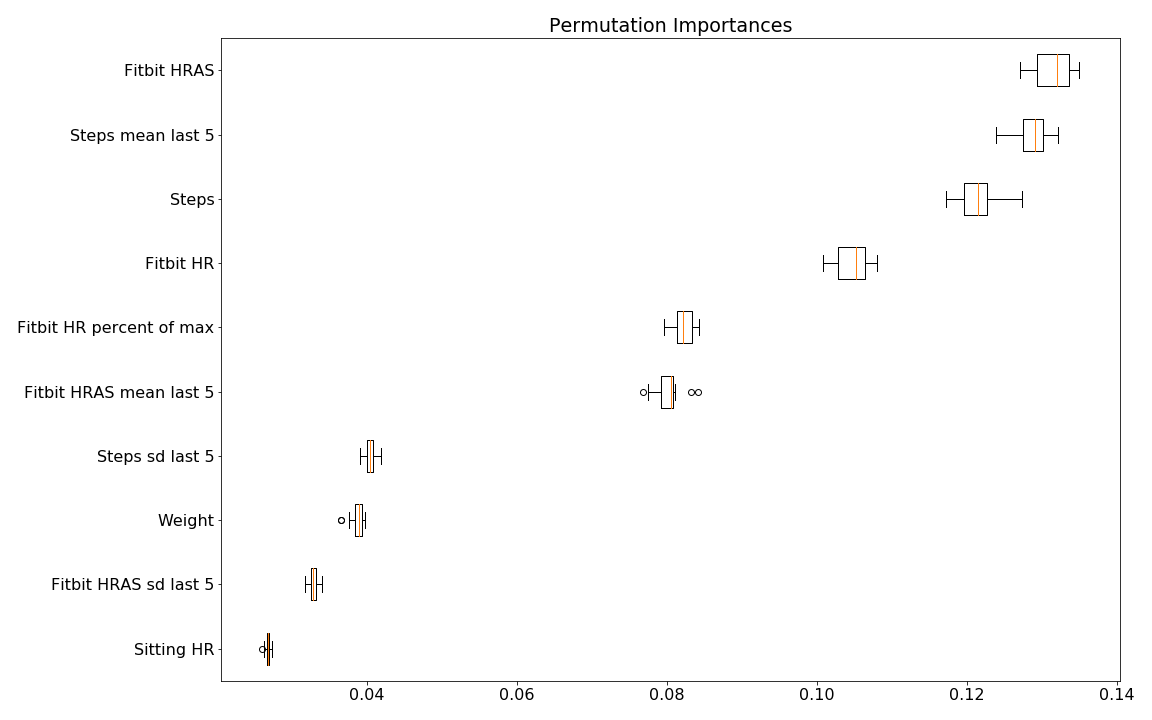
**

**Figure 1.** Permutation importance for the top 10 variables in the Fitbit dataset.

Abbreviations: Heart rate above sitting (HRAS), heart rate (HR), standard deviation (SD).

**SenseWear**

**
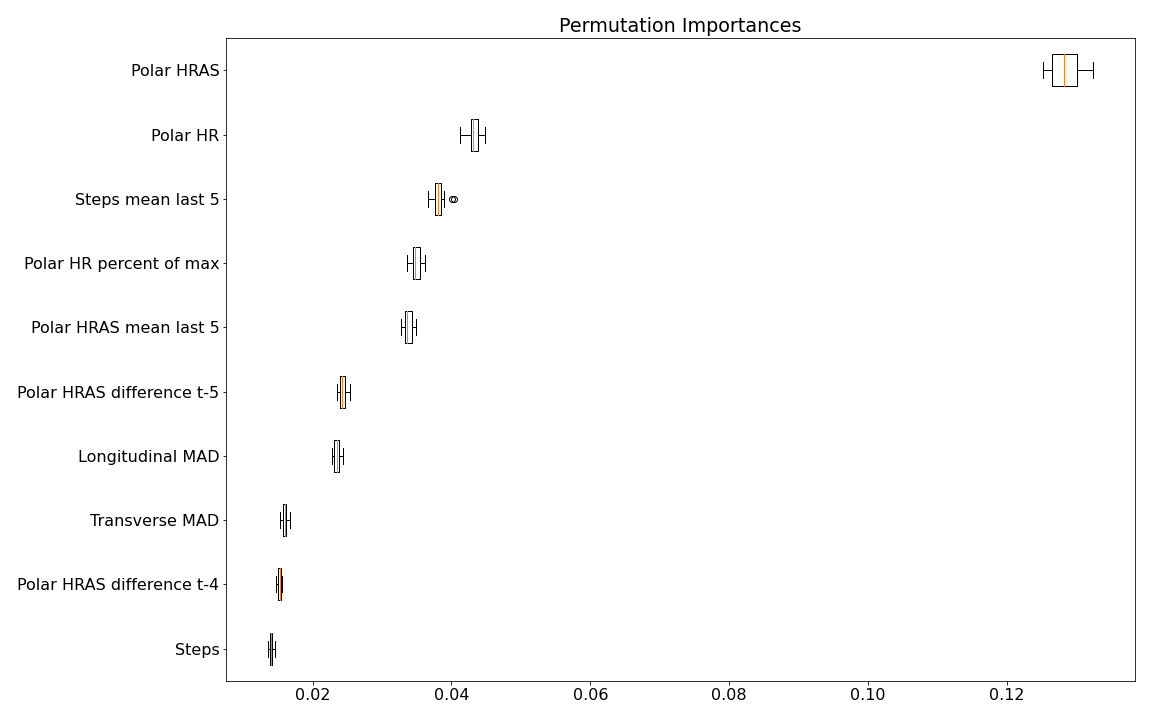
**

**Figure 2.** Permutation importance for the top 10 variables in the SenseWear dataset.

Abbreviations: Heart rate above sitting (HRAS), heart rate (HR), Mean of absolute differences (MAD).

**Actigraph**

**
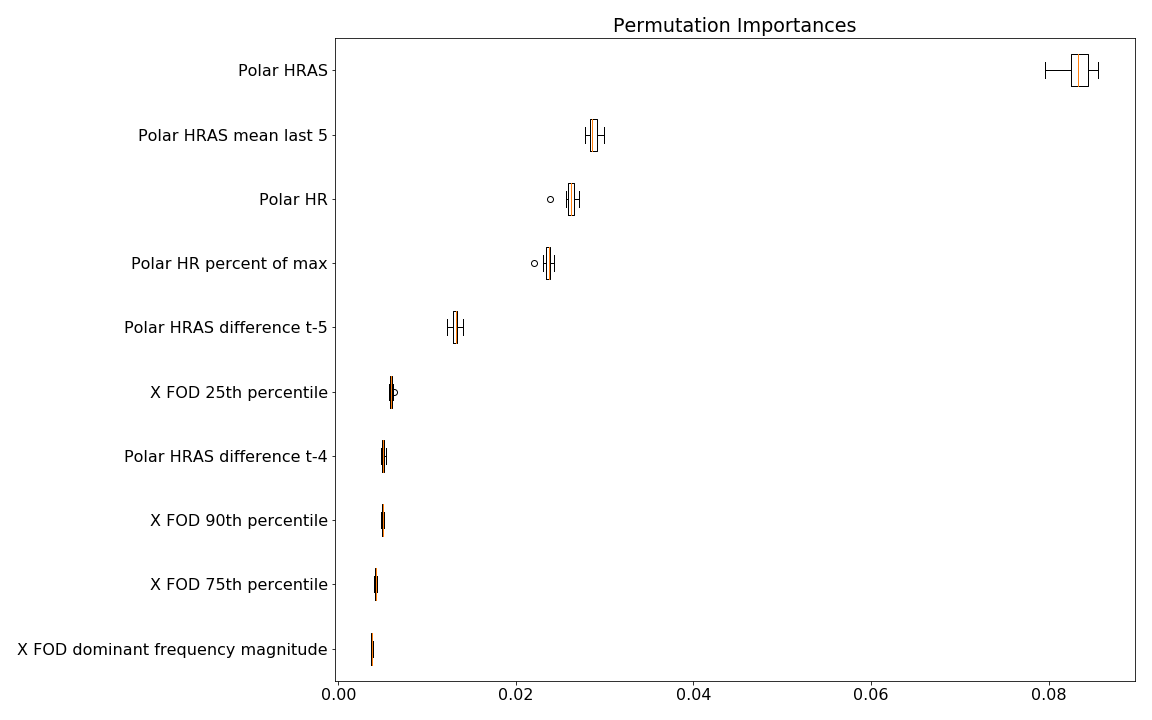
**

**Figure 3.** Permutation importance for the top 10 variables in the ActiGraph dataset.

Abbreviations: Heart rate above sitting (HRAS), heart rate (HR), X-Axis (X), first order differential (FOD).
