# Supplementary material for: Comparison of the Validity and Generalizability of Machine Learning Algorithms for the Prediction of Energy Expenditure: Validation Study
Source: JMIR Mhealth Uhealth. 2021 Aug 4;9(8):e23938. doi: 10.2196/23938 (PMC8374660; doi:10.2196/23938)
Supplement: Multimedia Appendix 5 [file mhealth_v9i8e23938_app5.docx]

**Multimedia Appendix 5.** Leave-one-subject-out cross-validation results for each of the classification models in each of the intensity categories.

|  | | **Model** | **Sensitivity** | **Specificity** | **Precision** | **F1** | **Balanced Accuracy** |
| --- | --- | --- | --- | --- | --- | --- | --- |
| **Sedentary** | **AG Gradient Boost** | | 0.84 | 0.95 | 0.81 | 0.83 | 0.90 |
|  | **AG K Nearest Neighbors** | | 0.85 | 0.94 | 0.78 | 0.81 | 0.90 |
|  | **AG Neural Network** | | 0.82 | 0.95 | 0.79 | 0.81 | 0.88 |
|  | **AG Random Forest** | | 0.85 | 0.95 | 0.81 | 0.83 | 0.90 |
|  | **AG Support Vector Machine** | | 0.39 | 0.98 | 0.85 | 0.54 | 0.69 |
|  | **FB Gradient Boost** | | 0.79 | 0.93 | 0.74 | 0.76 | 0.86 |
|  | **FB K Nearest Neighbors** | | 0.88 | 0.87 | 0.63 | 0.74 | 0.88 |
|  | **FB Neural Network** | | 0.59 | 0.94 | 0.72 | 0.65 | 0.77 |
|  | **FB Random Forest** | | 0.81 | 0.93 | 0.74 | 0.77 | 0.87 |
|  | **FB Support Vector Machine** | | 0.68 | 0.93 | 0.72 | 0.70 | 0.81 |
|  | **SWA** | | 0.81 | 0.90 | 0.67 | 0.74 | 0.86 |
|  | **SWA Gradient Boost** | | 0.84 | 0.96 | 0.82 | 0.83 | 0.90 |
|  | **SWA K Nearest Neighbors** | | 0.87 | 0.94 | 0.77 | 0.82 | 0.91 |
|  | **SWA Neural Network** | | 0.77 | 0.91 | 0.68 | 0.72 | 0.84 |
|  | **SWA Random Forest** | | 0.84 | 0.96 | 0.82 | 0.83 | 0.90 |
|  | **SWA Support Vector Machine** | | 0.75 | 0.95 | 0.79 | 0.77 | 0.85 |
|  |  | |  |  |  |  |  |
| **Light** | **AG Gradient Boost** | | 0.59 | 0.92 | 0.68 | 0.63 | 0.76 |
|  | **AG K Nearest Neighbors** | | 0.60 | 0.89 | 0.62 | 0.61 | 0.75 |
|  | **AG Neural Network** | | 0.62 | 0.90 | 0.63 | 0.62 | 0.76 |
|  | **AG Random Forest** | | 0.57 | 0.93 | 0.70 | 0.63 | 0.75 |
|  | **AG Support Vector Machine** | | 0.18 | 0.97 | 0.67 | 0.28 | 0.58 |
|  | **FB Gradient Boost** | | 0.40 | 0.91 | 0.55 | 0.46 | 0.65 |
|  | **FB K Nearest Neighbors** | | 0.25 | 0.94 | 0.53 | 0.34 | 0.59 |
|  | **FB Neural Network** | | 0.18 | 0.93 | 0.43 | 0.25 | 0.56 |
|  | **FB Random Forest** | | 0.41 | 0.92 | 0.59 | 0.48 | 0.66 |
|  | **FB Support Vector Machine** | | 0.32 | 0.91 | 0.52 | 0.39 | 0.62 |
|  | **SWA** | | 0.37 | 0.84 | 0.41 | 0.39 | 0.61 |
|  | **SWA Gradient Boost** | | 0.61 | 0.93 | 0.70 | 0.66 | 0.77 |
|  | **SWA K Nearest Neighbors** | | 0.59 | 0.92 | 0.67 | 0.63 | 0.75 |
|  | **SWA Neural Network** | | 0.13 | 0.95 | 0.42 | 0.20 | 0.54 |
|  | **SWA Random Forest** | | 0.60 | 0.93 | 0.70 | 0.65 | 0.76 |
|  | **SWA Support Vector Machine** | | 0.52 | 0.91 | 0.61 | 0.57 | 0.71 |
|  |  | |  |  |  |  |  |
| **MVPA** | **AG Gradient Boost** | | 0.94 | 0.86 | 0.91 | 0.92 | 0.90 |
|  | **AG K Nearest Neighbors** | | 0.90 | 0.88 | 0.91 | 0.91 | 0.89 |
|  | **AG Neural Network** | | 0.92 | 0.88 | 0.92 | 0.92 | 0.90 |
|  | **AG Random Forest** | | 0.95 | 0.85 | 0.90 | 0.92 | 0.90 |
|  | **AG Support Vector Machine** | | 0.99 | 0.34 | 0.68 | 0.80 | 0.66 |
|  | **FB Gradient Boost** | | 0.91 | 0.77 | 0.84 | 0.87 | 0.84 |
|  | **FB K Nearest Neighbors** | | 0.90 | 0.77 | 0.84 | 0.87 | 0.84 |
|  | **FB Neural Network** | | 0.96 | 0.55 | 0.74 | 0.84 | 0.75 |
|  | **FB Random Forest** | | 0.92 | 0.76 | 0.84 | 0.88 | 0.84 |
|  | **FB Support Vector Machine** | | 0.94 | 0.68 | 0.80 | 0.86 | 0.81 |
|  | **SWA** | | 0.82 | 0.79 | 0.85 | 0.83 | 0.80 |
|  | **SWA Gradient Boost** | | 0.95 | 0.88 | 0.91 | 0.93 | 0.91 |
|  | **SWA K Nearest Neighbors** | | 0.93 | 0.90 | 0.93 | 0.93 | 0.91 |
|  | **SWA Neural Network** | | 0.96 | 0.63 | 0.78 | 0.86 | 0.79 |
|  | **SWA Random Forest** | | 0.95 | 0.87 | 0.91 | 0.93 | 0.91 |
|  | **SWA Support Vector Machine** | | 0.94 | 0.81 | 0.88 | 0.91 | 0.88 |

LOSO results for each of the classification models. Abbreviations: Fitbit (FB), ActiGraph (AG), SenseWear (SWA).
